# Small area variation in child undernutrition across 640 districts and 543 parliamentary constituencies in India

**DOI:** 10.1038/s41598-021-83992-6

**Published:** 2021-02-25

**Authors:** Sunil Rajpal, Julie Kim, William Joe, Rockli Kim, S. V. Subramanian

**Affiliations:** 1 Institute of Health Management Research, IIHMR University, Jaipur, India; 2 Harvard Center for Population and Development Studies, Cambridge, MA USA; 3 Population Research Centre, Institute of Economic Growth, Delhi, India; 4 Interdisciplinary Program in Precision Public Health, Department of Public Health Sciences, Graduate School of Korea University, Seoul, South Korea; 5 Division of Health Policy and Management, College of Health Science, Korea University, Seoul, South Korea; 6 Department of Social and Behavioral Sciences, Harvard T.H. Chan School of Public Health, Boston, MA USA

**Keywords:** Health policy, Nutrition, Public health

## Abstract

In India, districts serve as central policy unit for program development, administration and implementation. The one-size-fits-all approach based on average prevalence estimates at the district level fails to capture the substantial small area variation. In addition to district average, heterogeneity within districts should be considered in policy design. The objective of this study was to quantify the extent of small area variation in child stunting, underweight and wasting across 36 states/Union Territories (UTs), 640 districts (and 543 PCs), and villages/blocks in India. We utilized the 4th round of Indian National Family Health Survey (NFHS-4) conducted in 2015–2016. The study population included 225,002 children aged 0–59 months whose height and weight information were available. Stunting was defined as height-for-age z-score below 2 SD from the World Health Organization child growth reference standards. Similarly, underweight and wasting were each defined as weight-for-age < -2 SD and weight-for-height < -2 SD from the age- and sex-specific medians. We adopted a four-level logistic regression model to partition the total variation in stunting, underweight and wasting. We computed precision-weighted prevalence of child anthropometric failures across districts and PCs as well as within-district/PC variation using standard deviation (SD) measures. For stunting, 56.4% (var: 0.237; SE: 0.008) of the total variation was attributed to villages/blocks, followed by 25.8% (var: 0.109; SE: 0.030) to states/UTs, and 17.7% (Var: 0.074; SE: 0.006) to districts. For underweight and wasting, villages/blocks accounted for 38.4% (var: 0.224; SE: 0.007) and 50% (var: 0.285; SE: 0.009), respectively, of the total contextual variance in India. Similar findings were shown in multilevel models incorporating PC as a geographical unit instead of districts. We found high positive correlations between mean prevalence and SD for stunting (r = 0.780, *p* < 0.001), underweight (r = 0.860, *p* < 0.001), and wasting (r = 0.857, *p* < 0.001) across all districts in India. A similar pattern of correlation was found for PCs. Within-district and within-PC variation are the primary source of variation for child malnutrition in India. Our results suggest the importance of considering heterogeneity within districts and PCs when planning and administering child nutrition policies.

## Introduction

Monitoring and evaluation of health status in developing countries are primarily based on prevalence estimates across macro policy units. States or provinces often serve as a principal unit of measurement, in addition to increasing efforts to use the second-level geography^1,2^. In India, *districts* are the sub-division of states that serve as central administrative unit. The *Transformation of Aspirational Districts* initiative, which identified 115 “aspirational” districts, exemplifies the significance of districts in policy administration in India. The Government of India selected these districts based on performance indicators across six developmental domains and prioritized allocation of human and financial resources to advance progress in aspirational districts^3^. Similarly, POSHAN *Abhiyaan,* India’s national nutrition flagship programme, has been rolled out in different phases based on district-level prevalence of child undernutrition^4,5^. Both of these programmes adopted district-level average for policy decision and resource allocation.

However, increasing volume of evidence suggests that district average may mask substantial socioeconomic inequity across smaller area in India. Recent studies using multilevel modeling have consistently reported large small area variation in diverse development and health outcomes^6–10^. For example, a recent study on women’s Body Mass Index (BMI) across 58 low- and middle-income countries found that between-individual differences contributed to nearly 80% of the total variation in BMI^6^. In India, inter-village variation accounts for the largest share of poverty and child sex ratio^7,8^. These results imply that districts with above average performance might have significant underlying degree of inequality.

Despite of the underlying socioeconomic heterogeneity within districts, little is known about the intra-district (i.e. inter-village/block) variation in child malnutrition outcomes. Child malnutrition has been a central health concern in India, as it attributed to 68.2% of under-five deaths in 2017^2^. Previous studies that characterized geographic inequality of childhood anthropometric indicators across states and districts revealed high burden in northern and central states^11–13^. Yet, the variation within state or smaller levels of geography has not been fully explored.

In addition to districts, Parliamentary Constituencies (PCs) in India are a comparable geographical unit to districts that serve a political role. The Members of Parliament (MPs), elected from 543 PCs, represent in the Lok Sabha, the lower house equivalent in India^14^. The MPs hold governmental authority on resource allocation and policy implementation at the national and PC levels. For instance, MPs have decision-making power to mobilize the funds from Member of Parliament Local Area Development (MPLAD) Scheme^15^. While the lack of political identifiers in nationally representative surveys has hampered presenting PC-level progress in population health and development, recent studies developed methodologies to link the Census and other surveys to PCs^16–18^. These methods enabled computation of PC-level estimates for child undernutrition^16,17^, yet within-PC variation has been neither investigated nor quantified.

In this paper, we aimed to characterize and measure the variation of child malnutrition across 36 states and Union Territories (UTs), 640 districts and 543 PCs, and small area (villages/bocks) in India. First, we performed a multilevel analysis to partition the variation in child undernutrition across these geographies. These estimates can be interpreted as the relative importance of each geographic unit in determining the variation in child undernutrition outcomes. Then, we computed village/block-specific precision-weighted estimates of child undernutrition. Using these estimates, we present not only the overall mean of children malnutrition across districts and PCs, but also the within-district and within-PC variation for the first time in terms of SD. Lastly, we presented the relationship between the mean and SD across districts and PCs to highlight the importance of considering small area inequality measures when defining highly burden areas.

## Data and methods

### Survey data and study population

We obtained the data from the fourth round of National Family Health Survey (NFHS-4) of India (equivalent to Demographic and Health Survey) conducted in 2015–2016. For the first time, NFHS-4 collected and provided information for all 640 districts nested in 29 states and 7 UTs of India. The NFHS-4 contained a sample of 247,743 living children aged 0–59 months. After excluding those with missing information on child’s height and weight (22,741 children), 225,002 children remained in the final analytic sample.

### Survey design

In NFHS-4, the households were selected from a two-stage cluster sampling frame. According to DHS sampling manual, a cluster was defined as a group of adjacent households which served as the primary sampling units (PSU) for the efficiency of field work. A cluster is an *Enumeration Area* with a measure of size equal to the number of households (or the population) in that area, mostly provided by the population census^19^.

In the first stage, villages and census enumeration blocks (hereafter referred as villages/blocks) were selected as PSUs for rural and urban areas, respectively. A household listing operation was then carried out by visiting each of the selected PSUs and listing all residential households. In the PSUs with more than 300 households were divided into segments of 100–150 households^19^. Therefore, in NFHS-4, a cluster was a PSU or a segment of PSU. The resulting list of households served as sampling frame for selection of households in the second stage. A fixed number of 22 households were selected from each PSU based on equal probability systematic sampling.

### Primary outcomes

The primary outcome variables were three anthropometric failures—stunting, underweight and wasting. We defined all three outcomes following the World Health Organization (WHO) child growth reference standards^20^. Stunting was defined as height-for-age z-scores less than − 2 SD, underweight as weight-for-age z-scores less than − 2SD, and wasting as weight-for-height z-scores less than − 2SD. Age- and sex-specific z-scores were computed using the raw height and weight collected by skilled and trained field investigators of the NFHS-4. For children aged between 24 and 59 months, standing height was taken, whereas recumbent length was measured for children younger than 24 months. The weight was measured by skilled health investigators using digital solar-powered scales along with adjustable short measuring boards.

### Statistical analyses

PC membership for each child was identified following a previously published methodology^17,18^. We adopted a four-level logistic model to partition the total variation in child undernutrition outcomes (*Y*) by four levels of child *i* (level-1), cluster *j* (level-2), district or PC *k* (level-3), and state/UT *l* (level-4): ${Y}_{ijkl }={\beta }_{0}+({u}_{0jkl}+{v}_{0kl}+{f}_{0l})$. In this model, ${u}_{0jkl}$, ${v}_{0kl}$, and ${f}_{0l}$ are residuals specific to cluster, district (or PC), and state, respectively. Each set of residuals are assumed to be normally distributed with a mean of 0 and a variance of ${u}_{0jkl}\sim N(0, {\sigma }_{{u}_{0}}^{2})$, ${v}_{0kl}\sim N(0, {\sigma }_{{v}_{0}}^{2})$, and ${f}_{0l}\sim N(0, {\sigma }_{{f}_{0}}^{2})$. The term ${\sigma }_{{u}_{0}}^{2}$, therefore, denotes within-district (or PC), between-village/block variation; ${\sigma }_{{v}_{0}}^{2}$ represents within-state, between-district (or PC) variation; and ${\sigma }_{{f}_{0}}^{2}$ is the between-state variation. Individual level variance is not directly estimated for binary outcomes and is instead assumed to come from a logistic distribution with a fixed variance of π^2^/3 or 3.29^20^.

Variance partitioning coefficient can be calculated to assess the significance of variability across different unit of *z*: ($\frac{{\sigma }_{z}^{2}}{{\sigma }_{{u}_{0}}^{2}+{\sigma }_{{v}_{0}}^{2}+{\sigma }_{{f}_{0}}^{2}+\uppi 2/3})\mathrm{x}100$. Multilevel modeling was performed in MLwiN 3.0 software program via Monte Carlo Markov Chain (MCMC) methods using Gibbs sampler with the default prior distributions of Iterated Generalized Least Squares (IGLS) estimations as starting values, a burn-in of 500 cycles, and monitoring of 5000 iterations of chains^21^.

From the multilevel logistic model, we generated village/block-specific precision-weighted estimates of child undernutrition, which were predicted from pooling information and borrowing strength from other villages/bocks that share the same district membership, for more reliable estimates^22,23^. The probability of *Y* for each village/bock was calculated as: exp(${\beta }_{0}+{u}_{0jkl}+{v}_{0kl}+{f}_{0l}$)/(1 + exp(${\beta }_{0}+{u}_{0jkl}+{v}_{0kl}+{f}_{0l}$)). From the above precision-weighted estimates of child undernutrition, we further estimated within-district (or within-PC) between-village/block variations in child stunting, underweight and wasting by computing SDs.

All maps were generated using ArcGIS Desktop 10.6. The shapefiles for 640 districts as per the year 2011 and 543 PCs as per the year 2014 were obtained from the Community Created Maps of India (CCMA) project (http://projects.datameet.org/maps/).

## Results

### Relative importance of districts and PCs

**Figure 1:**
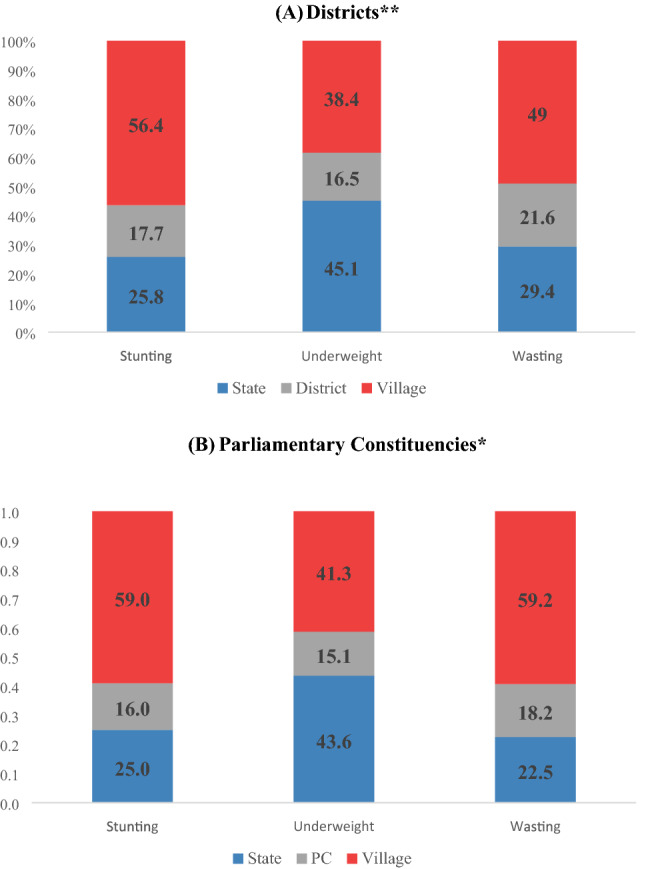
Partitioning variation in child anthropometric failures (0–59 months) by multiple geographies, India, NFHS 2016. *Note:* The exact variance estimates are reported in supplementary Table S1.

The variance partitioning estimates from multilevel logistic models indicated a relatively large proportion of variation in child anthropometric failures attributed to small area (villages and blocks in rural and urban areas, respectively) within a district or PC (Fig. 1). Of the total variance in stunting, 56.4% (var: 0.237; SE: 0.008) was attributed to villages/bocks, followed by 25.8% (var: 0.109; SE: 0.030) to states, and 17.7% (var: 0.074; SE: 0.006) to districts (Fig. 1A; Table S1). For underweight, about 45% of variation (var: 0.263; SE: 0.069) was at the state level, followed by 38.4% (var: 0.224; SE: 0.007) at village/block level, and 16.5% (var: 0.096; SE: 0.007) at district level. Villages/blocks accounted for about half (var: 0.285; SE: 0.009) of the total contextual variance in child wasting. We found similar patterns in multilevel models incorporating PC as a geographical unit instead of districts (Fig. 1B).

### Within-district small area variation in child undernutrition

**Figure 2:**
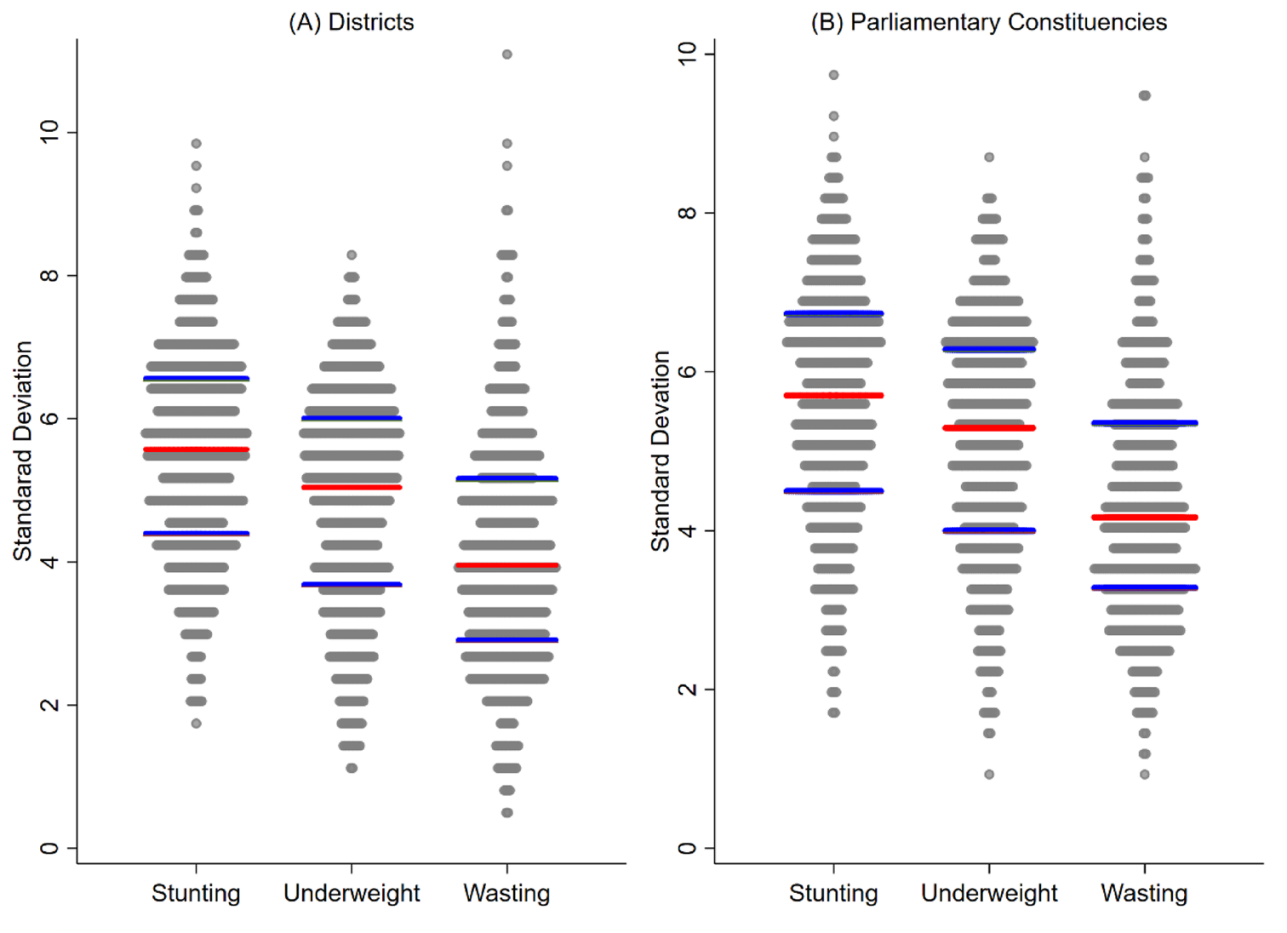
Dot plots showing distribution of standard deviations (SDs) of child anthropometric failures within (**A**) districts; (**B**) parliamentary constituencies. *Note*: Red lines represent median SD and blue lines represent 25th and 75th percentiles of SD.

**Figure 3:**
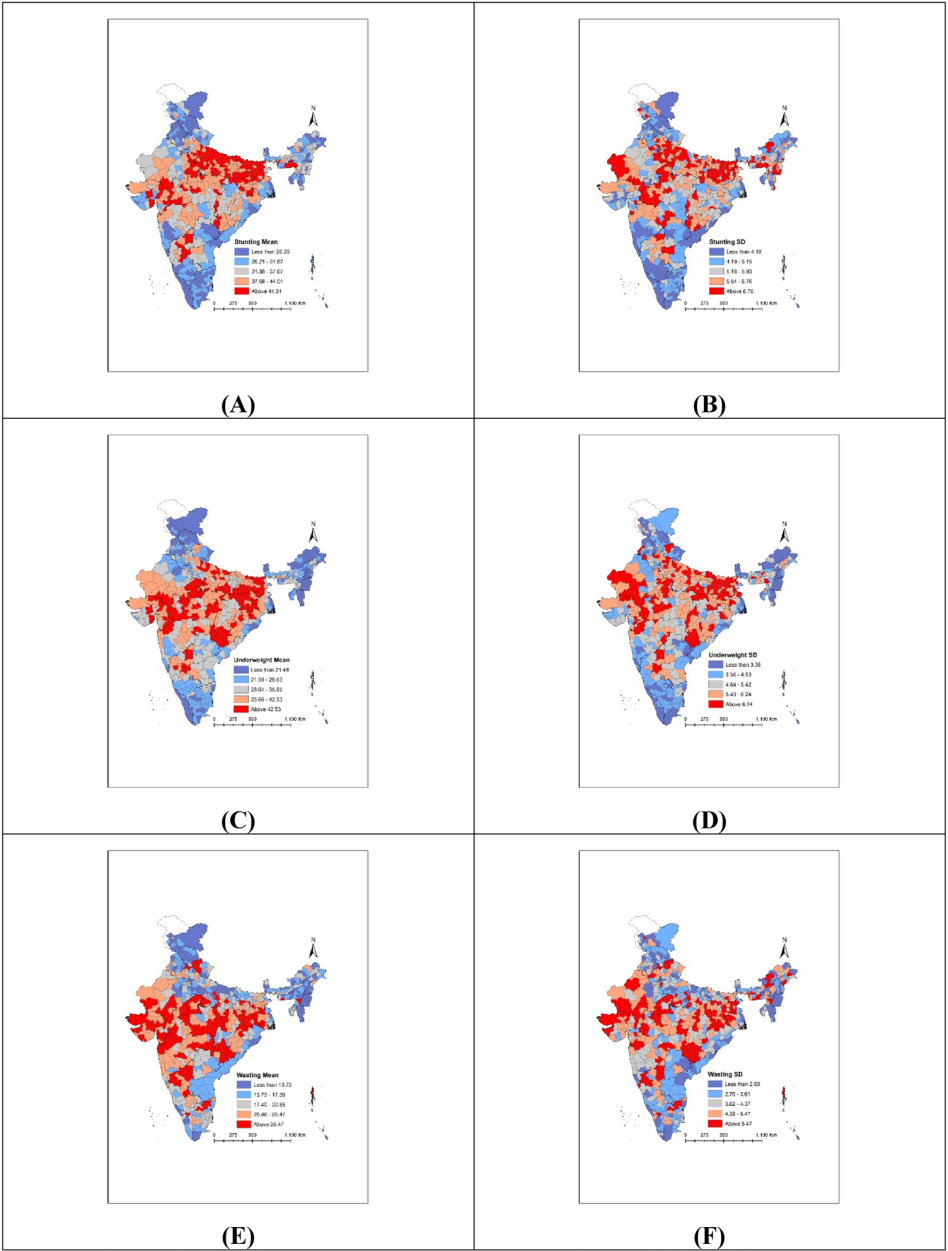
Maps showing geographic distribution of mean prevalence and SDs in child anthropometric failures across districts for (**A**) stunting—mean; (**B**) stunting—SD; (**C**) underweight—mean; (**D**) underweight—SD; (**E**) wasting—mean; (**F**) wasting—SD. *Note*: Figures were generated using the spatial join tools in ArcGIS Desktop 10.6 (http://desktop.arcgis.com/en/arcmap/latest/tools/analysis-toolbox/spatial-join.htm).

The within-district small area variation was measured by SD of stunting, underweight and wasting. Figure 2A shows that SD across districts ranged from 1.8 to 9.8 (IQR: 2.5) for stunting, 1.0–8.5 (IQR: 2.2) for underweight, and 0.9–8.8 (IQR: 1.9) for wasting. The range of SD across districts was relatively higher for wasting compared to stunting and underweight. However, the median value of SD across districts was observed to be the highest for stunting (5.6). Maps presented in Fig. 3 show the spatial distribution of predicted mean and between-village/block SD across districts. In case of stunting, high-burden districts (prevalence above the average 44.1%) are visibly concentrated in North-Eastern districts (colored in red) (Fig. 3A). Districts with large small area variation in stunting (between-village/block SD above 6.76) were also concentrated in similar regions (colored in red) (Fig. 3B). On the contrary, most of the southern districts had low-burden (below 21.4%) of child stunting (colored in blue) (Fig. 3A) as well as low level of SD (less than 3.35) (Fig. 3B). We found similar patterns in spatial distribution for underweight and wasting. For instance, clustering of high-burden districts for underweight (above 42.53%) was evident in central and eastern India (Fig. 3C). Interestingly, most of those high-burden districts for underweight also had substantial small area variation (SD above 6.24) (Fig. 3D).

### Within-PC small area variation in child undernutrition

**Figure 4:**
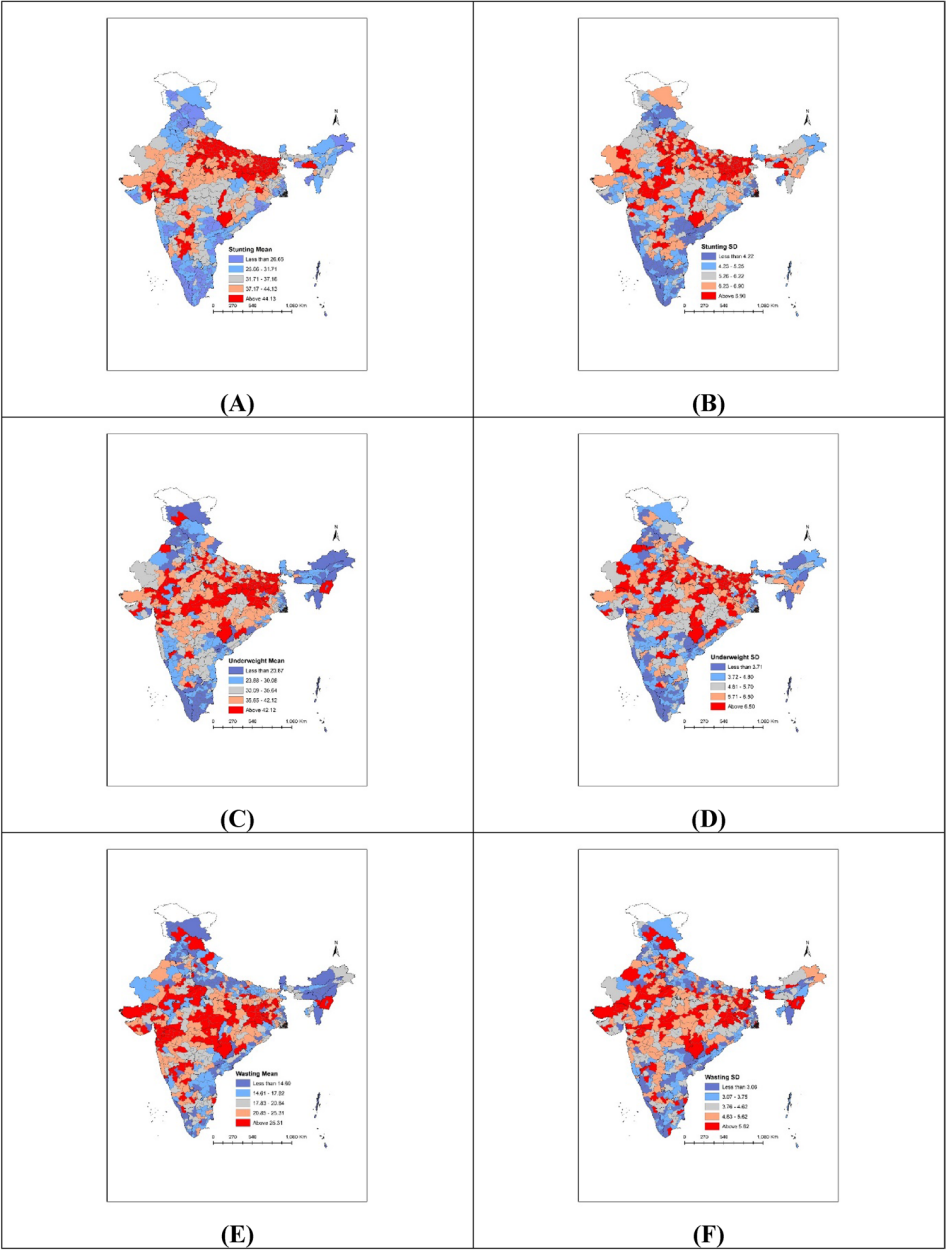
Maps showing geographic distribution of mean prevalence and SDs in child anthropometric failures across PCs for (**A**) stunting—mean; (**B**) stunting—SD; (**C**) underweight—mean; (**D**) underweight—SD; (**E**) wasting—mean; (**F**) wasting—SD. *Note*: Figures were generated using the spatial join tools in ArcGIS Desktop 10.6 (http://desktop.arcgis.com/en/arcmap/latest/tools/analysis-toolbox/spatial-join.htm).

Within-PC small area variation (measured as between-village/block SD) in child stunting ranged from 1.9 to 9.8 with the median of 5.5 (Fig. 2B). In case of underweight and wasting, the median values of between-cluster SD across PCs were 5.2 and 4.2 respectively. Compared to underweight and wasting, distribution of between-cluster SD across PCs was relatively higher for stunting. Maps presented in Fig. 4 depict similar spatial distribution of mean prevalence of and SD in child anthropometric failures across PCs. For instance, North-Eastern region had high burden of stunting (colored in red) in Fig. 4A. The between-cluster SD in stunting was also higher for most of the PCs in North-Eastern region (Fig. 4B). Similarly, in case of underweight (Fig. 4C,D), PCs in the lowest quintile of mean prevalence tended to have lower SD. Similar pattern was observed for wasting (Fig. 4E,F).

### Correlation between mean prevalence and small area variation

**Figure 5:**
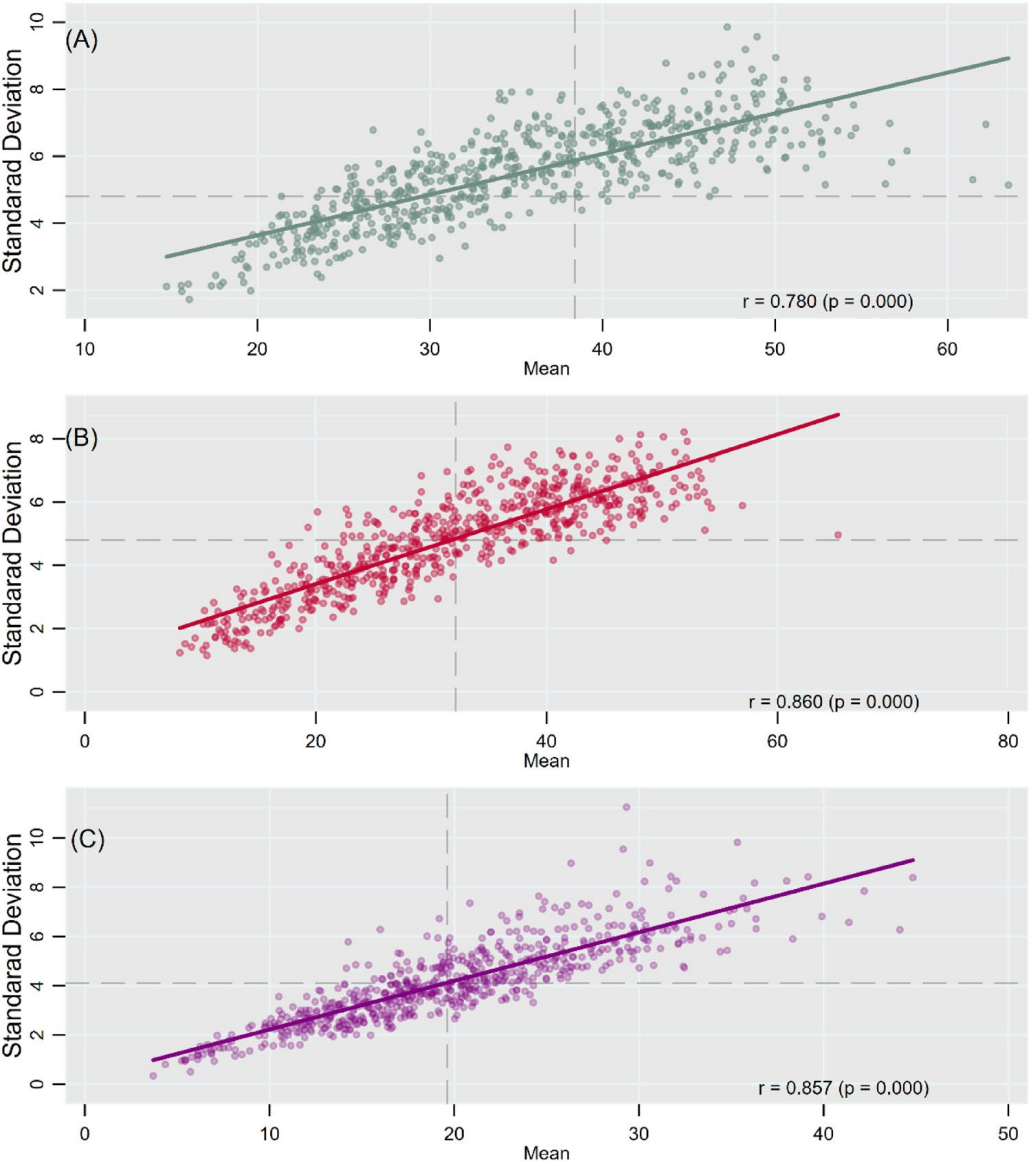
Scatter plots showing correlation between mean and SD of child anthropometric failures across districts for (**A**) stunting; (**b**) underweight; (**C**) wasting, India, NFHS 2016.

Figure 5A demonstrates a strong positive correlation between mean prevalence and between-village/block SD in stunting across all districts in India (r = 0.780, *p* < 0.001). About 41% (264 districts) of the districts were clustered in the quadrant of high mean and high SD and in the quadrant of low mean and low SD. Similarly, a high and positive correlation was observed between mean prevalence of underweight and SD across all districts (r = 0.860, *p* < 0.001; Fig. 5B). For child wasting, the correlation coefficient between mean prevalence and SD across districts was 0.857 (*p* < 0.001; Fig. 5C).

**Figure 6:**
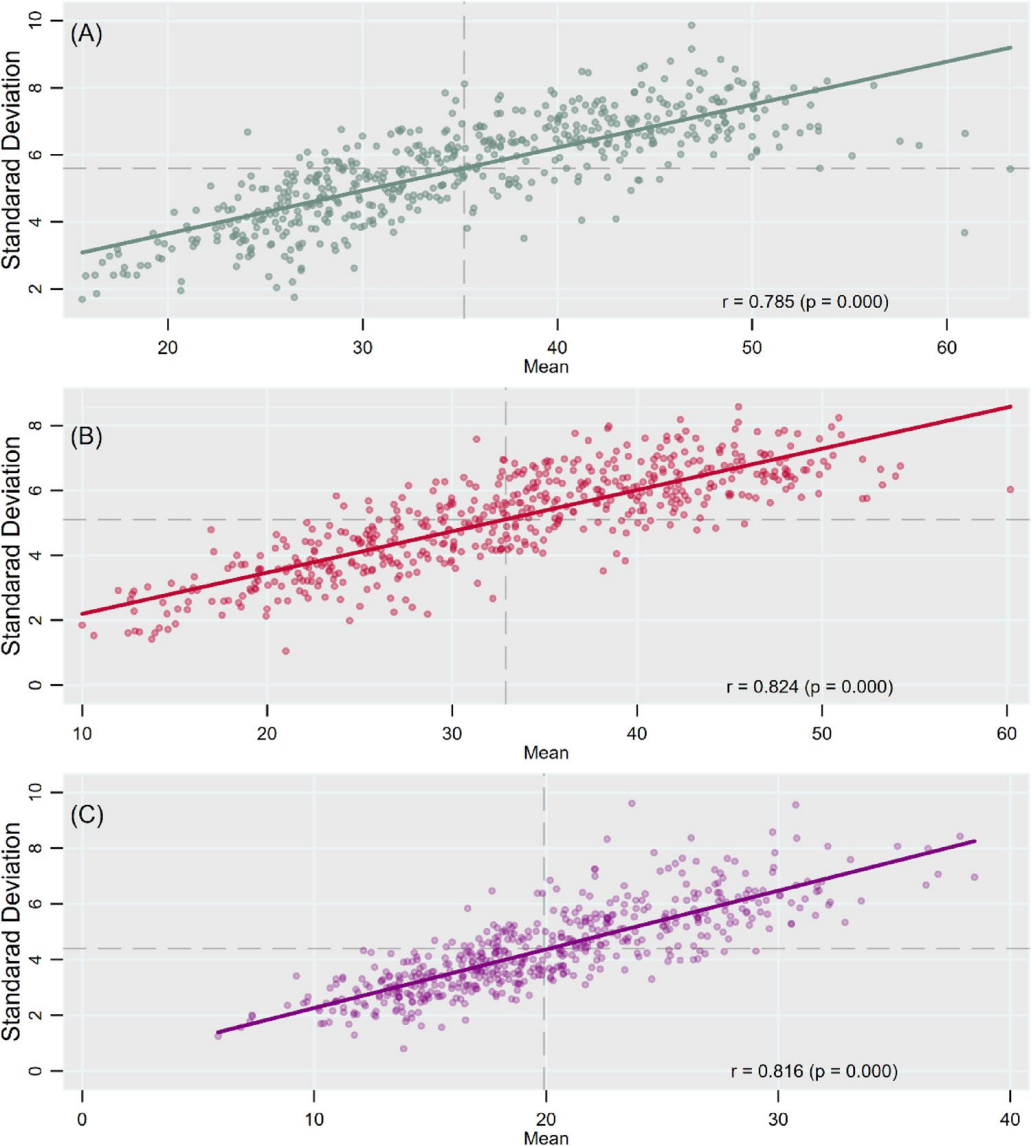
Scatter Plots showing correlation between mean and SD of child anthropometric failures across PCs for (**A**) stunting; (**B**) underweight; (**C**) wasting, India, NFHS 2016.

We found a consistently high and positive correlation between mean prevalence and within-PC between-village/block variation in anthropometric failures (Fig. 6). For example, for child stunting, the correlation was high and positive across all PCs (r = 0.785, *p* < 0.001; Fig. 6A). In the same vein, a high degree of positive correlation between prevalence and between-village/block variation across PCs was observed for child underweight (r = 0.824, *p* < 0.001; Fig. 6B) and wasting (r = 0.816, *p* < 0.001; Fig. 6C).

## Discussion

We aimed to quantify and examine variation in child anthropometric failures across four important geographical levels: states/UTs, district, PCs, and villages/blocks in India. Three salient findings emerged from our analysis. First, among the multiple geographies considered, small area variation within districts (and PCs) accounted for the largest share of the total variance in child undernutrition. This finding on the substantial small area variation is consistent with findings from prior multilevel studies on the socioeconomic and health inequities across villages in India^9–12,24,25^. Second, we quantified within-PC variation in child undernutrition for the first time. Similar to the results from districts, villages/blocks accounted for the largest variation across all geographies. Third, we showed a strong and positive correlation between mean prevalence for districts (or PCs) and within-district (or within-PC) variation in child stunting, underweight and wasting. This implies that districts and PCs with higher overall burden also tend to have larger small area variation. These findings could guide policy design in several aspects.

First, this information could contribute to selecting an appropriate geographic unit to serve as a building block for effective administration, governance, monitoring, and evaluation. In a populous country like India with limited resources, widespread developmental concerns, and geographical and socio-cultural heterogeneities, identifying the most relevant geographic policy units are crucial for designing interventions. Despite such relevance, existing data, academic research, and policy discourse on development and health in India largely rely on average performances of districts. This masks the potential utility of other important policy units and the existence of substantial small area variation underlying within districts.

Second, our findings provide integral information for the current initiatives in India to reduce child undernutrition. For example, ongoing programs such as POSHAN *Abhiyaan* focus on average district-level prevalence and performances^4^. However, the results from variance partitioning suggest the need to consider small area variation when selecting policy units for action. This finding is critical to attain desired reductions in the mean prevalence and underlying inequality of child anthropometric failures. For example, Bilaspur district of Chhattisgarh have mean prevalence of child stunting of 34.05% which is below the national mean prevalence. However, within Bilaspur, substantial small area variation (SD: 7.89) was observed with the mean prevalence ranging from < 1 to 75% across villages/blocks. In this case, district average may mask the specific needs of different villages or municipal wards in Bilaspur. To identify high-burden villages/blocks within districts, health programmes should incorporate inequality metrics in performance evaluation.

Third, we observed high intra-PC inequality in child undernutrition across villages/blocks. The estimates from our multilevel model suggest that villages/blocks accounted for a relatively higher share in the total variation of child anthropometric failures. This finding possesses direct political utility as MPs hold governmental authority in resource allocation decisions. For example, MPs also raise funds by collaborating with several NGOs and think tanks. Given such authority, a detailed understanding of the within-PC variation is essential. By providing intra-PC variation estimates, we aimed to provide a monitoring framework that can potentially improve governmental accountability in reducing the burden of child malnutrition. In this regard, a few recent studies have also emphasized the use of political units as a monitoring framework to enhance evidence-based discourse among policymakers^16,17^. The PC-level estimates presented in this study could be further utilized to target smaller geographic areas to address pressing public health agenda.

Fourth, the strong and positive correlation between mean prevalence and inter-village/block variations (SD) across districts and PCs suggest the importance of considering inequality within districts. In this study, we showed that districts and PCs with higher overall burden are also subject to higher inequality. For instance, mean prevalence of stunting in Punch district of Jammu and Kashmir is 26.69% with high values of SD (6.78%). Although current strategy in India is to identify target-areas based on district-level averages of health status, we argue that considering small area variation within districts and PCs can further enhance efficiency by reducing the risk of missing high-burden areas nested within otherwise well-performing districts (or PCs).

This study has a few limitations. While we focus on villages and blocks to capture small area variation, rural villages and urban blocks are not directly comparable in terms of size and administrative role. Further, our precision-weighted estimates for mean prevalence and inter-village/block SD across districts and PCs do not adjust for demographic and socioeconomic correlates that may be driving higher burden of child undernutrition for some districts and PCs.

In conclusion, our findings suggest that health and nutritional inequality exists across smaller geographic area, which calls for more precise policy attention. The results presented in this paper could be utilized to determine whether it is more appropriate to target the general population or subpopulation in specific regions in order to improve the effectiveness of ongoing initiatives related to child malnutrition in India. The analyses conducted in this paper could be applied to other developing countries that share similar task of reducing the burden of child malnutrition.

## Supplementary Information

Supplementary Information
