# Supplementary material for: Small area variation in child undernutrition across 640 districts and 543 parliamentary constituencies in India
Source: Sci Rep. 2021 Feb 25;11:4558. doi: 10.1038/s41598-021-83992-6 (PMC7907088; doi:10.1038/s41598-021-83992-6)
Supplement: Supplementary file 1 — Supplementary Information [file 41598_2021_83992_MOESM1_ESM.docx]

**Supplementary**

**Small area variation in child undernutrition across 640 districts and 543 parliamentary constituencies in India**

**Sunil Rajpal, Julie Kim, William Joe, Rockli Kim, S.V. Subramanian**

Sunil Rajpal, Institute of Health Management Research, IIHMR University, Jaipur, India (sunilrajpal27@gmail.com).

Julie Kim, Harvard Center for Population and Development Studies, Cambridge, Massachusetts, USA (e-mail: [sojinkim@hsph.harvard.edu](mailto:sojinkim@hsph.harvard.edu)).

William Joe, Population Research Centre, Institute of Economic Growth, Delhi, India (william@iegindia.org)

Rockli Kim*, Interdisciplinary Program in Precision Public Health, Department of Public Health Sciences, Graduate School of Korea University, Seoul, South Korea;

Division of Health Policy and Management, College of Health Science, Korea University, Seoul, South Korea (e-mail: rocklikim@korea.ac.kr).

S.V. Subramanian*, Harvard Center for Population and Development Studies, Cambridge, Massachusetts, USA. Department of Social and Behavioral Sciences, Harvard T.H. Chan School of Public Health, Boston, Massachusetts, USA (e-mail: svsubram@hsph.harvard.edu).

***Co-corresponding authors**

Rockli Kim*, ScD, Division of Health Policy & Management, College of Health Science, Korea University, 145 Anam-ro, Seongbuk-gu, Seoul 02841, South Korea (e-mail: [rocklikim@korea.ac.kr](mailto:rocklikim@korea.ac.kr)).

S.V. Subramanian*, PhD, Harvard Center for Population and Development Studies, 9 Bow Street, Cambridge, MA 02138, USA (e-mail: svsubram@hsph.harvard.edu).

**Table S1.** Variation in Child Anthropometric Failures (0-59 months) by Multiple Geographies, India, NFHS 2016

| **Overall Mean** | **Stunting** | **Underweight** | **Wasting** |
| --- | --- | --- | --- |
| **Variance partitioning*** |  |  |  |
| State | 0.106 *(0.030)* | 0.251 *(0.075)* | 0.118 *(0.038)* |
| PC | 0.068 *(0.006)* | 0.087 *(0.007)* | 0.095 *(0.008)* |
| Village | 0.250 *(0.007)* | 0.237 *(0.008)* | 0.309 *(0.009)* |
| **Variance partitioning**** |  |  |  |
| State | 0.109 *(0.030)* | 0.263 *(0.069)* | 0.171 *(0.050)* |
| District | 0.074 *(0.006)* | 0.096 *(0.007)* | 0.126 *(0.009)* |
| Village | 0.237 *(0.008)* | 0.224 *(0.007)* | 0.285 *(0.009)* |

*Four-level Logistic Regression Model with Village-PC-State-Individual level Random Effects

** Four-level Logistic Regression Model with Village-District-State-Individual level Random Effects

**Table S2:** Mean Prevalence and Standard Deviation of Child Anthropometric Failures by Districts, India, NFHS, 2016

|  | **Stunting** | | **Underweight** | | **Wasting** | |
| --- | --- | --- | --- | --- | --- | --- |
| **Districts** | **Mean** | **SD** | **Mean** | **SD** | **Mean** | **SD** |
| Adilabad | 33.98 | 6.18 | 33.04 | 5.01 | 19.83 | 3.39 |
| Agra | 44.03 | 7.25 | 34.53 | 5.52 | 13.76 | 3.25 |
| Ahmadabad | 28.98 | 3.59 | 32.14 | 4.35 | 25.95 | 4.59 |
| Ahmadnagar | 34.56 | 4.24 | 31.81 | 4.69 | 21.80 | 4.04 |
| Aizawl | 23.37 | 4.45 | 8.22 | 1.24 | 3.70 | 0.34 |
| Ajmer | 32.67 | 5.58 | 37.39 | 6.59 | 29.49 | 7.27 |
| Akola | 34.74 | 5.50 | 35.66 | 5.08 | 21.75 | 4.16 |
| Alappuzha | 15.58 | 2.14 | 16.86 | 2.22 | 15.47 | 1.99 |
| Aligarh | 46.67 | 7.03 | 37.46 | 6.21 | 15.21 | 3.81 |
| Alirajpur | 47.45 | 8.76 | 51.13 | 7.58 | 31.61 | 7.94 |
| Allahabad | 43.91 | 6.45 | 42.66 | 6.55 | 18.72 | 3.72 |
| Almora | 31.38 | 4.74 | 22.73 | 3.59 | 15.17 | 3.20 |
| Alwar | 40.83 | 7.53 | 34.26 | 5.62 | 18.14 | 3.95 |
| Ambala | 21.27 | 4.21 | 32.03 | 5.72 | 35.32 | 9.82 |
| Ambedkar nagar | 42.91 | 6.64 | 39.64 | 5.98 | 20.85 | 3.86 |
| Amravati | 37.72 | 4.87 | 32.48 | 5.11 | 21.63 | 3.60 |
| Amreli | 36.71 | 5.25 | 31.66 | 4.90 | 22.81 | 3.45 |
| Amritsar | 21.67 | 4.24 | 13.69 | 2.57 | 11.16 | 2.27 |
| Anand | 44.34 | 5.82 | 38.50 | 5.34 | 21.33 | 3.85 |
| Anantapur | 37.67 | 6.58 | 37.60 | 5.88 | 15.36 | 3.11 |
| Anantnag | 19.73 | 3.65 | 9.23 | 1.42 | 6.11 | 1.01 |
| Anjaw | 23.25 | 4.14 | 12.06 | 1.73 | 14.37 | 2.21 |
| Anugul | 30.09 | 5.79 | 33.24 | 5.14 | 20.60 | 3.60 |
| Anuppur | 33.61 | 5.75 | 39.44 | 5.03 | 29.67 | 5.37 |
| Araria | 48.01 | 6.86 | 45.34 | 6.41 | 21.74 | 5.34 |
| Ariyalur | 32.80 | 4.34 | 26.82 | 3.85 | 17.57 | 2.79 |
| Arwal | 49.23 | 7.86 | 52.98 | 6.47 | 29.14 | 9.54 |
| Ashoknagar | 42.50 | 6.99 | 46.49 | 6.57 | 29.80 | 6.15 |
| Auraiya | 42.25 | 5.45 | 43.82 | 6.09 | 24.99 | 5.88 |
| Aurangabad | 47.80 | 6.77 | 46.24 | 5.92 | 23.62 | 4.98 |
| Aurangabad | 36.81 | 6.07 | 35.17 | 4.40 | 20.84 | 4.17 |
| Azamgarh | 40.35 | 6.64 | 32.81 | 4.84 | 16.51 | 3.62 |
| Badgam | 21.81 | 4.03 | 10.36 | 2.57 | 5.23 | 0.95 |
| Bagalkot | 46.36 | 5.86 | 42.06 | 5.39 | 23.35 | 3.51 |
| Bageshwar | 25.19 | 4.83 | 25.26 | 3.70 | 23.92 | 5.08 |
| Baghpat | 34.74 | 6.15 | 33.41 | 5.09 | 13.97 | 2.60 |
| Bahraich | 63.53 | 5.14 | 42.67 | 7.11 | 12.76 | 3.78 |
| Baksa | 32.10 | 5.28 | 22.21 | 2.68 | 10.49 | 2.44 |
| Balaghat | 33.19 | 4.49 | 41.49 | 5.42 | 31.16 | 5.51 |
| Balangir | 41.50 | 6.00 | 42.59 | 5.80 | 24.30 | 6.21 |
| Baleshwar | 32.29 | 4.72 | 32.62 | 4.85 | 17.33 | 3.42 |
| Ballia | 39.63 | 6.66 | 31.71 | 5.85 | 14.51 | 2.96 |
| Balrampur | 62.22 | 6.95 | 42.99 | 6.52 | 10.27 | 2.24 |
| Banaskantha | 39.88 | 6.73 | 43.30 | 6.42 | 21.96 | 6.17 |
| Banda | 45.14 | 6.06 | 40.60 | 5.36 | 17.32 | 2.87 |
| Bandipore | 21.39 | 4.25 | 10.66 | 2.14 | 6.84 | 1.38 |
| Bangalore | 28.58 | 3.92 | 26.66 | 3.83 | 25.48 | 4.79 |
| Bangalore rural | 30.56 | 2.95 | 28.30 | 2.98 | 22.56 | 3.41 |
| Banka | 50.14 | 7.28 | 48.50 | 5.78 | 24.58 | 4.86 |
| Bankura | 32.77 | 5.39 | 38.34 | 6.69 | 25.00 | 5.85 |
| Banswara | 48.22 | 6.84 | 48.91 | 7.43 | 28.95 | 5.36 |
| Bara banki | 50.23 | 7.35 | 39.70 | 6.45 | 12.26 | 2.69 |
| Baramula | 28.78 | 5.05 | 13.15 | 3.25 | 12.46 | 3.35 |
| Baran | 38.80 | 4.39 | 39.07 | 5.86 | 27.39 | 5.49 |
| Barddhaman | 31.30 | 5.24 | 32.11 | 5.06 | 23.45 | 4.20 |
| Bareilly | 44.00 | 6.77 | 39.67 | 6.24 | 17.30 | 3.86 |
| Bargarh | 37.20 | 5.11 | 37.49 | 5.29 | 23.15 | 3.77 |
| Barmer | 36.59 | 6.97 | 39.18 | 5.94 | 25.62 | 6.29 |
| Barnala | 23.57 | 4.10 | 16.90 | 2.28 | 11.92 | 2.20 |
| Barpeta | 39.96 | 4.85 | 31.48 | 4.91 | 15.15 | 3.32 |
| Barwani | 50.59 | 6.77 | 54.31 | 7.36 | 26.89 | 7.32 |
| Bastar | 40.40 | 6.64 | 48.51 | 6.63 | 31.73 | 6.86 |
| Basti | 48.34 | 7.02 | 33.31 | 5.51 | 13.49 | 2.90 |
| Bathinda | 23.45 | 4.23 | 17.11 | 2.58 | 10.28 | 2.49 |
| Baudh | 39.38 | 6.87 | 41.27 | 5.88 | 21.12 | 3.34 |
| Begusarai | 44.65 | 6.98 | 38.37 | 7.29 | 18.01 | 4.18 |
| Belgaum | 34.94 | 5.46 | 37.05 | 4.44 | 29.55 | 5.98 |
| Bellary | 47.51 | 5.90 | 48.67 | 6.41 | 24.02 | 4.99 |
| Betul | 34.88 | 5.32 | 44.65 | 5.07 | 32.46 | 4.73 |
| Bhadrak | 34.10 | 6.53 | 28.34 | 4.74 | 14.42 | 2.73 |
| Bhagalpur | 46.77 | 8.27 | 41.54 | 6.94 | 23.53 | 6.95 |
| Bhandara | 35.52 | 5.12 | 30.05 | 4.25 | 17.27 | 3.53 |
| Bharatpur | 45.59 | 7.15 | 29.90 | 5.49 | 14.76 | 3.34 |
| Bharuch | 39.62 | 5.87 | 41.61 | 5.35 | 26.65 | 4.79 |
| Bhavnagar | 46.21 | 4.80 | 43.19 | 5.02 | 25.16 | 4.68 |
| Bhilwara | 34.51 | 4.88 | 40.57 | 4.16 | 33.43 | 5.95 |
| Bhind | 46.07 | 7.00 | 49.25 | 6.94 | 29.79 | 6.89 |
| Bhiwani | 34.03 | 7.70 | 26.51 | 4.89 | 15.35 | 3.54 |
| Bhojpur | 43.65 | 6.98 | 48.04 | 6.60 | 26.32 | 8.97 |
| Bhopal | 46.61 | 6.76 | 39.84 | 6.36 | 20.46 | 4.02 |
| Bid | 35.96 | 5.48 | 36.79 | 5.25 | 28.43 | 4.92 |
| Bidar | 42.50 | 5.06 | 37.97 | 5.09 | 22.85 | 5.24 |
| Bijapur | 46.68 | 8.74 | 46.30 | 8.00 | 24.78 | 7.11 |
| Bijapur | 42.21 | 6.67 | 35.59 | 4.93 | 26.92 | 4.74 |
| Bijnor | 42.99 | 7.57 | 41.36 | 6.87 | 21.56 | 5.42 |
| Bikaner | 32.38 | 5.79 | 31.46 | 6.19 | 23.40 | 5.58 |
| Bilaspur | 26.34 | 4.52 | 21.36 | 4.01 | 11.47 | 2.10 |
| Bilaspur | 34.05 | 7.89 | 33.59 | 6.58 | 25.80 | 7.00 |
| Birbhum | 38.47 | 5.80 | 40.60 | 5.91 | 26.99 | 5.84 |
| Bishnupur | 25.61 | 4.58 | 13.46 | 2.49 | 6.40 | 1.21 |
| Bokaro | 38.88 | 6.65 | 49.77 | 7.15 | 36.34 | 6.31 |
| Bongaigaon | 37.21 | 6.77 | 30.23 | 5.31 | 19.95 | 5.13 |
| Budaun | 54.67 | 6.84 | 53.05 | 6.19 | 19.68 | 3.89 |
| Bulandshahr | 41.94 | 7.59 | 32.71 | 6.26 | 15.12 | 3.73 |
| Buldana | 40.99 | 6.36 | 39.84 | 5.68 | 21.01 | 3.30 |
| Bundi | 37.19 | 5.76 | 40.92 | 5.17 | 25.48 | 5.10 |
| Burhanpur | 46.18 | 7.41 | 42.30 | 7.44 | 18.21 | 5.01 |
| Buxar | 43.68 | 8.78 | 41.12 | 7.62 | 18.83 | 4.70 |
| Cachar | 34.89 | 5.18 | 34.76 | 5.42 | 28.30 | 6.05 |
| Central | 32.04 | 3.31 | 30.59 | 2.94 | 20.07 | 3.12 |
| Chamarajanagar | 31.21 | 3.81 | 31.66 | 4.08 | 19.04 | 3.40 |
| Chamba | 28.19 | 4.77 | 20.77 | 4.61 | 13.15 | 3.08 |
| Chamoli | 32.88 | 4.76 | 22.80 | 2.96 | 17.05 | 3.91 |
| Champawat | 29.48 | 6.72 | 21.70 | 3.68 | 16.88 | 3.68 |
| Champhai | 30.68 | 5.98 | 11.53 | 2.54 | 5.46 | 0.98 |
| Chandauli | 43.25 | 6.94 | 35.23 | 7.14 | 16.58 | 4.38 |
| Chandel | 36.19 | 6.85 | 13.74 | 3.12 | 6.64 | 1.61 |
| Chandigarh | 29.52 | 3.46 | 24.00 | 3.89 | 10.64 | 2.18 |
| Chandrapur | 31.81 | 4.87 | 38.12 | 4.68 | 26.36 | 4.75 |
| Changlang | 24.38 | 4.42 | 18.84 | 3.41 | 14.69 | 3.82 |
| Chatra | 49.37 | 7.11 | 49.51 | 6.23 | 28.38 | 7.13 |
| Chennai | 30.76 | 5.78 | 22.10 | 4.00 | 19.54 | 4.72 |
| Chhatarpur | 41.18 | 5.77 | 40.74 | 5.16 | 18.84 | 3.74 |
| Chhindwara | 33.95 | 5.94 | 40.09 | 4.91 | 28.95 | 5.07 |
| Chikkaballapura | 36.30 | 4.90 | 30.53 | 4.22 | 17.72 | 3.57 |
| Chikmagalur | 23.70 | 2.38 | 26.01 | 3.56 | 20.39 | 3.67 |
| Chirang | 36.87 | 6.82 | 22.85 | 5.29 | 12.15 | 3.01 |
| Chitradurga | 30.58 | 4.68 | 31.35 | 4.79 | 30.01 | 4.72 |
| Chitrakoot | 50.34 | 7.95 | 51.90 | 8.21 | 31.72 | 8.43 |
| Chittaurgarh | 37.03 | 5.49 | 40.10 | 6.21 | 21.90 | 4.93 |
| Chittoor | 29.87 | 5.56 | 31.38 | 5.10 | 16.33 | 3.42 |
| Churachandpur | 34.33 | 7.67 | 10.25 | 2.14 | 6.10 | 1.20 |
| Churu | 30.44 | 5.61 | 27.50 | 4.52 | 20.85 | 4.87 |
| Coimbatore | 25.74 | 4.52 | 22.71 | 3.58 | 20.40 | 3.53 |
| Cuddalore | 28.27 | 4.97 | 24.86 | 4.24 | 18.00 | 3.89 |
| Cuttack | 17.83 | 2.13 | 19.27 | 2.67 | 10.76 | 1.88 |
| Dadra & nagar haveli | 40.64 | 6.73 | 36.98 | 5.81 | 25.42 | 5.03 |
| Dakshin bastar dantewada | 41.40 | 6.32 | 49.05 | 6.44 | 29.38 | 5.55 |
| Dakshin dinajpur | 34.00 | 5.90 | 27.77 | 3.97 | 16.59 | 2.79 |
| Dakshina kannada | 25.00 | 3.03 | 23.26 | 2.54 | 17.62 | 2.85 |
| Daman | 22.16 | 2.95 | 25.13 | 3.66 | 24.26 | 4.32 |
| Damoh | 42.69 | 6.07 | 36.88 | 5.40 | 20.34 | 4.02 |
| Darbhanga | 48.92 | 7.57 | 40.76 | 6.74 | 15.16 | 3.38 |
| Darjiling | 29.05 | 4.06 | 26.11 | 4.32 | 12.21 | 2.21 |
| Darrang | 41.07 | 7.97 | 35.43 | 7.42 | 18.17 | 3.66 |
| Datia | 46.84 | 6.17 | 45.44 | 5.31 | 24.28 | 4.01 |
| Dausa | 33.02 | 5.67 | 27.94 | 4.55 | 15.80 | 3.82 |
| Davanagere | 44.99 | 6.39 | 40.75 | 5.73 | 20.95 | 3.15 |
| Debagarh | 31.77 | 4.78 | 36.14 | 5.04 | 19.04 | 3.75 |
| Dehradun | 27.82 | 4.55 | 30.73 | 5.03 | 28.61 | 5.87 |
| Deoghar | 44.44 | 6.83 | 45.65 | 6.25 | 22.61 | 4.18 |
| Deoria | 42.41 | 7.75 | 31.86 | 5.51 | 13.28 | 2.66 |
| Dewas | 40.01 | 7.52 | 42.64 | 6.41 | 24.79 | 5.33 |
| Dhalai | 29.70 | 4.80 | 25.21 | 4.26 | 21.16 | 3.95 |
| Dhamtari | 34.53 | 5.39 | 40.23 | 5.62 | 25.86 | 4.64 |
| Dhanbad | 38.64 | 5.14 | 41.96 | 5.35 | 27.86 | 5.35 |
| Dhar | 42.85 | 6.89 | 48.49 | 6.80 | 30.34 | 5.87 |
| Dharmapuri | 24.58 | 2.97 | 26.68 | 4.53 | 30.36 | 6.13 |
| Dharwad | 38.52 | 4.90 | 42.67 | 6.07 | 32.78 | 5.87 |
| Dhaulpur | 52.58 | 6.73 | 38.97 | 6.03 | 15.61 | 2.87 |
| Dhemaji | 34.87 | 6.03 | 16.53 | 2.10 | 7.00 | 0.94 |
| Dhenkanal | 25.38 | 4.94 | 29.30 | 4.23 | 18.79 | 3.66 |
| Dhubri | 45.53 | 7.23 | 36.93 | 5.99 | 19.67 | 5.74 |
| Dhule | 35.48 | 6.63 | 42.71 | 6.49 | 29.54 | 5.39 |
| Dibang valley | 32.56 | 4.65 | 13.82 | 1.46 | 11.01 | 2.00 |
| Dibrugarh | 32.35 | 4.26 | 30.53 | 5.55 | 19.32 | 4.69 |
| Dima hasao | 34.04 | 5.99 | 18.72 | 3.70 | 7.30 | 1.68 |
| Dimapur | 21.32 | 3.44 | 13.64 | 2.29 | 7.77 | 1.49 |
| Dindigul | 28.91 | 4.99 | 26.59 | 4.53 | 23.06 | 4.92 |
| Dindori | 45.24 | 5.72 | 45.83 | 5.91 | 25.93 | 5.36 |
| Diu | 32.44 | 5.04 | 28.58 | 4.71 | 16.34 | 2.53 |
| Doda | 34.53 | 4.99 | 17.57 | 3.32 | 11.64 | 2.88 |
| Dohad | 42.55 | 5.35 | 49.79 | 5.94 | 23.54 | 4.24 |
| Dumka | 41.69 | 6.14 | 51.22 | 5.95 | 39.15 | 8.42 |
| Dungarpur | 45.84 | 6.48 | 52.07 | 7.40 | 35.14 | 7.10 |
| Durg | 33.11 | 5.67 | 34.65 | 5.98 | 19.86 | 4.12 |
| East | 24.45 | 3.44 | 21.50 | 2.36 | 19.94 | 3.37 |
| East district | 25.76 | 3.61 | 11.76 | 1.89 | 11.72 | 2.36 |
| East garo hills | 30.60 | 5.26 | 18.12 | 4.00 | 13.04 | 3.17 |
| East godavari | 27.61 | 3.60 | 26.91 | 4.21 | 14.32 | 2.77 |
| East kameng | 40.42 | 6.93 | 20.83 | 3.36 | 14.25 | 5.78 |
| East khasi hills | 46.13 | 7.56 | 28.75 | 4.82 | 10.94 | 2.72 |
| East siang | 21.24 | 2.75 | 16.25 | 2.42 | 18.09 | 3.76 |
| Ernakulam | 16.00 | 2.18 | 13.70 | 1.88 | 13.78 | 2.31 |
| Erode | 25.59 | 5.42 | 16.36 | 2.67 | 14.58 | 3.49 |
| Etah | 49.56 | 5.58 | 32.35 | 5.51 | 10.01 | 2.42 |
| Etawah | 51.92 | 6.95 | 33.23 | 6.05 | 11.99 | 2.88 |
| Faizabad | 50.55 | 5.95 | 44.87 | 6.08 | 17.78 | 3.95 |
| Faridabad | 30.93 | 4.39 | 25.18 | 4.20 | 22.46 | 5.78 |
| Faridkot | 31.28 | 6.00 | 25.26 | 5.35 | 16.92 | 4.07 |
| Farrukhabad | 47.59 | 7.07 | 30.91 | 4.87 | 9.03 | 2.39 |
| Fatehabad | 27.98 | 4.26 | 29.71 | 5.55 | 20.37 | 3.96 |
| Fatehgarh sahib | 20.31 | 3.68 | 18.15 | 3.34 | 14.15 | 2.23 |
| Fatehpur | 51.56 | 6.35 | 39.80 | 5.69 | 14.51 | 2.66 |
| Firozabad | 43.45 | 6.38 | 27.14 | 4.82 | 10.67 | 2.84 |
| Firozpur | 26.00 | 4.42 | 29.14 | 6.32 | 21.65 | 5.27 |
| Gadag | 33.74 | 6.16 | 36.23 | 5.83 | 38.32 | 5.90 |
| Gadchiroli | 32.40 | 4.79 | 42.81 | 5.28 | 44.12 | 6.27 |
| Gajapati | 31.69 | 4.50 | 31.63 | 5.31 | 17.93 | 4.31 |
| Ganderbal | 24.55 | 5.02 | 9.55 | 1.70 | 5.42 | 0.94 |
| Gandhinagar | 34.74 | 5.99 | 41.06 | 6.51 | 28.20 | 4.81 |
| Ganganagar | 29.83 | 4.65 | 28.10 | 4.47 | 19.38 | 4.94 |
| Ganjam | 29.67 | 5.34 | 23.03 | 3.70 | 16.50 | 2.44 |
| Garhwa | 45.85 | 6.68 | 50.97 | 6.33 | 29.68 | 6.43 |
| Garhwal | 24.56 | 3.70 | 26.73 | 3.58 | 26.04 | 5.33 |
| Gautam buddha nagar | 32.70 | 6.74 | 27.52 | 5.58 | 13.73 | 3.16 |
| Gaya | 51.86 | 8.28 | 52.17 | 7.92 | 24.56 | 5.26 |
| Ghaziabad | 34.34 | 6.37 | 28.67 | 5.26 | 12.30 | 2.42 |
| Ghazipur | 41.71 | 4.83 | 31.90 | 3.63 | 17.42 | 4.13 |
| Giridih | 47.64 | 7.74 | 42.36 | 6.49 | 23.37 | 6.21 |
| Goalpara | 41.45 | 7.85 | 36.34 | 6.85 | 19.19 | 6.72 |
| Godda | 51.80 | 5.65 | 45.68 | 5.18 | 24.70 | 6.18 |
| Golaghat | 32.27 | 5.53 | 20.64 | 3.36 | 13.31 | 3.30 |
| Gonda | 56.40 | 5.17 | 38.11 | 5.47 | 9.57 | 1.51 |
| Gondiya | 34.33 | 4.83 | 39.29 | 5.05 | 28.00 | 4.63 |
| Gopalganj | 37.49 | 5.76 | 32.12 | 5.39 | 16.28 | 3.72 |
| Gorakhpur | 41.82 | 6.63 | 34.63 | 5.51 | 18.37 | 3.68 |
| Gulbarga | 48.26 | 7.15 | 51.97 | 6.46 | 31.55 | 6.24 |
| Gumla | 46.52 | 4.98 | 48.40 | 5.97 | 30.22 | 5.93 |
| Guna | 42.62 | 7.44 | 50.18 | 5.47 | 31.99 | 6.34 |
| Guntur | 23.81 | 3.63 | 28.68 | 3.74 | 16.55 | 2.68 |
| Gurdaspur | 22.13 | 3.77 | 19.59 | 3.23 | 14.34 | 3.70 |
| Gurgaon | 35.74 | 6.86 | 28.58 | 5.13 | 19.43 | 4.95 |
| Gwalior | 42.07 | 6.36 | 47.40 | 6.47 | 27.84 | 6.02 |
| Hailakandi | 36.86 | 7.19 | 31.03 | 5.37 | 17.65 | 3.83 |
| Hamirpur | 28.06 | 3.59 | 18.76 | 2.61 | 12.16 | 2.54 |
| Hamirpur | 40.54 | 6.27 | 39.93 | 5.62 | 30.11 | 6.74 |
| Hanumangarh | 33.89 | 6.16 | 23.80 | 4.03 | 19.71 | 5.14 |
| Haora | 32.53 | 5.01 | 28.02 | 3.73 | 15.48 | 3.08 |
| Harda | 38.14 | 6.56 | 39.30 | 6.21 | 24.31 | 4.95 |
| Hardoi | 49.83 | 6.77 | 39.95 | 5.91 | 15.33 | 3.33 |
| Hardwar | 38.06 | 6.20 | 24.47 | 4.44 | 12.09 | 2.70 |
| Hassan | 27.18 | 3.57 | 25.94 | 3.15 | 19.19 | 2.88 |
| Haveri | 41.13 | 5.61 | 34.39 | 4.37 | 18.97 | 3.80 |
| Hazaribagh | 47.81 | 7.49 | 46.75 | 6.85 | 24.29 | 5.14 |
| Hingoli | 37.70 | 4.71 | 36.07 | 4.15 | 22.72 | 4.55 |
| Hisar | 25.89 | 4.46 | 24.42 | 4.59 | 22.84 | 6.64 |
| Hoshangabad | 34.46 | 5.68 | 38.18 | 5.71 | 28.94 | 5.42 |
| Hoshiarpur | 25.71 | 3.79 | 20.04 | 2.87 | 14.98 | 2.75 |
| Hugli | 28.99 | 4.37 | 27.79 | 3.98 | 17.53 | 3.42 |
| Hyderabad | 20.84 | 3.93 | 22.59 | 3.97 | 18.31 | 3.79 |
| Idukki | 16.05 | 1.72 | 13.96 | 1.54 | 20.29 | 3.36 |
| Imphal east | 25.35 | 4.19 | 15.57 | 3.15 | 6.97 | 1.45 |
| Imphal west | 21.39 | 4.80 | 8.68 | 1.52 | 4.35 | 0.81 |
| Indore | 37.95 | 6.38 | 31.12 | 4.94 | 19.12 | 4.75 |
| Jabalpur | 36.50 | 5.44 | 44.80 | 6.14 | 30.44 | 5.50 |
| Jagatsinghapur | 20.91 | 3.05 | 18.55 | 2.00 | 13.04 | 2.29 |
| Jaintia hills | 48.66 | 8.60 | 32.79 | 6.55 | 15.35 | 4.84 |
| Jaipur | 33.01 | 5.12 | 23.73 | 3.96 | 12.49 | 2.56 |
| Jaisalmer | 36.14 | 7.17 | 35.96 | 7.20 | 20.94 | 5.27 |
| Jajapur | 30.08 | 5.56 | 29.79 | 5.34 | 16.08 | 3.03 |
| Jalandhar | 28.02 | 4.40 | 22.68 | 3.38 | 15.90 | 3.12 |
| Jalaun | 45.50 | 5.15 | 47.52 | 5.28 | 29.68 | 5.08 |
| Jalgaon | 32.41 | 6.34 | 35.04 | 5.07 | 30.79 | 4.83 |
| Jalna | 41.57 | 6.15 | 41.18 | 5.85 | 21.54 | 4.46 |
| Jalor | 43.88 | 6.48 | 41.36 | 6.93 | 24.89 | 7.41 |
| Jalpaiguri | 31.03 | 4.29 | 25.89 | 3.06 | 17.36 | 3.00 |
| Jammu | 22.82 | 3.52 | 14.55 | 2.88 | 12.01 | 3.56 |
| Jamnagar | 29.74 | 5.21 | 30.27 | 3.74 | 30.30 | 8.23 |
| Jamtara | 44.39 | 6.49 | 48.03 | 6.85 | 28.66 | 5.73 |
| Jamui | 45.78 | 8.44 | 45.69 | 7.44 | 27.03 | 6.61 |
| Janjgir - champa | 34.83 | 5.64 | 34.97 | 4.05 | 22.26 | 4.34 |
| Jashpur | 34.51 | 5.95 | 34.25 | 6.39 | 17.54 | 4.63 |
| Jaunpur | 47.51 | 6.10 | 51.60 | 7.22 | 26.02 | 6.32 |
| Jehanabad | 50.24 | 7.63 | 45.27 | 6.76 | 18.92 | 4.19 |
| Jhabua | 44.98 | 7.39 | 45.18 | 6.69 | 24.84 | 5.50 |
| Jhajjar | 23.30 | 4.14 | 21.32 | 4.00 | 14.84 | 3.18 |
| Jhalawar | 37.81 | 5.46 | 45.91 | 6.56 | 29.47 | 5.91 |
| Jhansi | 35.90 | 5.62 | 38.92 | 5.07 | 25.97 | 5.94 |
| Jharsuguda | 32.69 | 5.19 | 34.74 | 4.78 | 23.38 | 3.83 |
| Jhunjhunun | 32.41 | 6.44 | 20.99 | 4.03 | 14.23 | 2.41 |
| Jind | 25.70 | 4.75 | 28.57 | 5.15 | 25.44 | 7.17 |
| Jodhpur | 39.95 | 6.56 | 37.86 | 6.49 | 22.19 | 4.38 |
| Jorhat | 26.70 | 3.74 | 19.87 | 2.90 | 13.95 | 3.06 |
| Junagadh | 31.15 | 4.49 | 28.64 | 4.74 | 26.42 | 5.16 |
| Jyotiba phule nagar | 43.09 | 7.36 | 40.60 | 6.43 | 21.01 | 3.84 |
| Kabirdham | 39.05 | 6.62 | 38.09 | 6.61 | 17.81 | 4.12 |
| Kachchh | 38.34 | 6.38 | 38.08 | 6.02 | 32.02 | 8.25 |
| Kaimur (bhabua) | 53.63 | 6.76 | 48.04 | 5.65 | 21.47 | 5.56 |
| Kaithal | 33.35 | 6.61 | 37.04 | 6.62 | 23.52 | 5.47 |
| Kalahandi | 35.13 | 5.69 | 37.90 | 6.18 | 22.76 | 3.52 |
| Kamrup | 33.27 | 6.13 | 28.10 | 5.65 | 17.41 | 4.24 |
| Kamrup metropolitan | 25.60 | 3.64 | 22.10 | 3.49 | 11.15 | 1.57 |
| Kancheepuram | 24.38 | 4.58 | 16.42 | 2.06 | 14.02 | 2.25 |
| Kandhamal | 34.96 | 5.93 | 39.77 | 6.28 | 21.15 | 3.77 |
| Kangra | 24.64 | 5.01 | 22.09 | 4.25 | 11.64 | 1.60 |
| Kannauj | 49.62 | 6.35 | 32.71 | 4.91 | 11.57 | 2.84 |
| Kanniyakumari | 19.34 | 2.71 | 15.00 | 2.26 | 10.82 | 2.33 |
| Kannur | 22.35 | 3.18 | 12.58 | 1.51 | 11.60 | 2.10 |
| Kanpur dehat | 45.34 | 5.57 | 36.54 | 5.77 | 15.35 | 3.11 |
| Kanpur nagar | 43.87 | 5.41 | 41.07 | 6.11 | 21.96 | 5.12 |
| Kanshiram nagar | 50.62 | 5.97 | 33.03 | 5.33 | 11.49 | 2.51 |
| Kapurthala | 21.79 | 3.96 | 19.39 | 3.61 | 15.46 | 3.88 |
| Karaikal | 19.45 | 2.66 | 23.02 | 2.95 | 20.79 | 3.83 |
| Karauli | 43.49 | 5.50 | 35.11 | 6.72 | 17.66 | 3.94 |
| Karbi anglong | 29.04 | 5.79 | 23.88 | 4.93 | 16.95 | 5.33 |
| Kargil | 37.64 | 6.63 | 19.18 | 3.41 | 7.31 | 1.47 |
| Karimganj | 39.44 | 6.40 | 34.17 | 5.35 | 17.10 | 4.63 |
| Karimnagar | 25.01 | 4.21 | 25.65 | 2.86 | 19.36 | 3.30 |
| Karnal | 39.23 | 6.85 | 31.37 | 6.96 | 18.35 | 4.71 |
| Karur | 25.42 | 5.14 | 26.69 | 3.86 | 20.71 | 4.01 |
| Kasaragod | 18.01 | 2.23 | 14.36 | 1.37 | 10.72 | 1.62 |
| Kathua | 29.51 | 5.41 | 17.56 | 2.76 | 13.72 | 3.22 |
| Katihar | 48.55 | 7.14 | 44.50 | 6.10 | 19.76 | 4.13 |
| Katni | 42.89 | 6.20 | 41.92 | 4.86 | 23.62 | 4.35 |
| Kaushambi | 49.13 | 5.91 | 51.53 | 6.53 | 28.65 | 6.46 |
| Kendrapara | 26.21 | 4.65 | 24.16 | 4.03 | 12.95 | 2.27 |
| Kendujhar | 42.13 | 6.44 | 42.81 | 6.79 | 18.84 | 3.69 |
| Khagaria | 48.78 | 7.86 | 41.33 | 7.49 | 16.80 | 4.08 |
| Khammam | 26.08 | 3.21 | 22.31 | 2.62 | 13.78 | 2.36 |
| Khandwa (east nimar) | 42.79 | 6.89 | 46.30 | 6.07 | 21.38 | 4.29 |
| Khargone (west nimar) | 48.41 | 7.26 | 44.47 | 6.05 | 20.74 | 3.50 |
| Kheda | 44.43 | 5.23 | 46.22 | 5.52 | 26.04 | 4.92 |
| Kheri | 53.15 | 7.55 | 40.05 | 5.69 | 17.44 | 4.96 |
| Khordha | 23.04 | 3.76 | 19.46 | 3.27 | 13.26 | 2.77 |
| Khunti | 40.50 | 6.23 | 53.63 | 6.31 | 42.19 | 7.84 |
| Kinnaur | 20.53 | 2.69 | 16.01 | 1.87 | 11.49 | 2.35 |
| Kiphire | 36.10 | 7.23 | 23.91 | 4.30 | 14.15 | 4.71 |
| Kishanganj | 47.01 | 7.42 | 45.14 | 6.12 | 22.57 | 4.91 |
| Kishtwar | 28.54 | 4.89 | 17.69 | 4.63 | 14.66 | 4.85 |
| Koch bihar | 31.98 | 5.66 | 28.74 | 4.26 | 19.29 | 4.51 |
| Kodagu | 30.49 | 4.40 | 25.56 | 4.01 | 16.36 | 2.80 |
| Kodarma | 44.02 | 5.87 | 43.34 | 6.31 | 19.82 | 4.15 |
| Kohima | 25.50 | 3.72 | 16.02 | 2.34 | 11.88 | 2.75 |
| Kokrajhar | 30.71 | 5.56 | 25.85 | 5.01 | 14.57 | 3.04 |
| Kolar | 31.97 | 4.79 | 28.05 | 3.22 | 17.83 | 2.77 |
| Kolasib | 26.98 | 4.92 | 10.94 | 2.17 | 6.11 | 1.33 |
| Kolhapur | 27.23 | 4.34 | 30.24 | 4.27 | 21.62 | 4.30 |
| Kolkata | 27.79 | 3.90 | 23.21 | 2.98 | 18.14 | 3.36 |
| Kollam | 14.73 | 2.11 | 13.25 | 1.56 | 17.23 | 3.64 |
| Koppal | 54.42 | 6.62 | 47.31 | 6.75 | 24.86 | 4.91 |
| Koraput | 37.84 | 5.96 | 42.40 | 5.54 | 27.54 | 5.17 |
| Korba | 31.60 | 5.60 | 34.64 | 5.21 | 23.79 | 4.45 |
| Korea (koriya) | 27.04 | 4.71 | 31.21 | 4.81 | 27.47 | 5.61 |
| Kota | 31.23 | 5.35 | 37.96 | 5.97 | 26.41 | 5.18 |
| Kottayam | 19.94 | 3.38 | 12.20 | 1.59 | 15.55 | 2.84 |
| Kozhikode | 17.33 | 2.13 | 16.92 | 2.28 | 13.65 | 2.29 |
| Krishna | 23.48 | 2.48 | 26.07 | 3.28 | 17.15 | 3.14 |
| Krishnagiri | 23.43 | 3.71 | 21.56 | 3.82 | 18.72 | 2.81 |
| Kulgam | 22.64 | 4.27 | 11.22 | 2.18 | 7.81 | 1.79 |
| Kullu | 19.10 | 3.08 | 13.07 | 1.93 | 12.11 | 2.40 |
| Kupwara | 30.06 | 6.06 | 14.81 | 2.51 | 6.25 | 1.54 |
| Kurnool | 41.19 | 7.29 | 34.71 | 4.59 | 16.70 | 2.92 |
| Kurukshetra | 30.38 | 6.03 | 26.72 | 5.78 | 22.55 | 5.46 |
| Kurung kumey | 37.55 | 7.68 | 25.39 | 5.30 | 20.84 | 7.35 |
| Kushinagar | 45.45 | 6.62 | 36.04 | 6.50 | 14.58 | 3.38 |
| Lahul and spiti | 22.67 | 2.81 | 17.01 | 2.12 | 17.31 | 2.73 |
| Lakhimpur | 29.00 | 5.55 | 23.14 | 4.92 | 10.53 | 2.99 |
| Lakhisarai | 49.09 | 8.19 | 45.41 | 5.89 | 19.30 | 5.19 |
| Lakshadweep | 26.33 | 3.66 | 21.50 | 3.18 | 12.82 | 2.09 |
| Lalitpur | 41.51 | 6.35 | 48.13 | 5.71 | 35.64 | 7.55 |
| Latehar | 43.97 | 6.32 | 44.26 | 5.93 | 27.99 | 5.48 |
| Latur | 33.66 | 5.34 | 33.86 | 5.50 | 21.45 | 5.48 |
| Lawngtlai | 33.07 | 5.88 | 19.33 | 5.20 | 10.50 | 3.56 |
| Leh | 20.20 | 3.58 | 16.68 | 4.05 | 12.93 | 3.45 |
| Lohardaga | 41.58 | 5.46 | 47.72 | 5.70 | 27.42 | 5.32 |
| Lohit | 27.03 | 5.78 | 22.40 | 4.94 | 19.75 | 5.94 |
| Longleng | 27.14 | 5.32 | 16.90 | 3.21 | 16.57 | 4.04 |
| Lower dibang valley | 23.57 | 3.94 | 15.29 | 2.61 | 19.71 | 4.98 |
| Lower subansiri | 31.11 | 4.32 | 19.90 | 2.94 | 20.31 | 3.32 |
| Lucknow | 40.32 | 4.66 | 43.97 | 4.94 | 29.91 | 5.47 |
| Ludhiana | 25.68 | 5.18 | 25.14 | 4.91 | 16.95 | 3.83 |
| Lunglei | 23.53 | 3.73 | 11.62 | 1.96 | 5.77 | 1.10 |
| Madhepura | 50.88 | 7.58 | 48.04 | 7.08 | 23.51 | 6.80 |
| Madhubani | 51.88 | 8.04 | 45.16 | 5.70 | 18.16 | 4.74 |
| Madurai | 22.33 | 3.57 | 20.05 | 3.23 | 14.05 | 2.03 |
| Mahamaya nagar | 43.80 | 7.13 | 31.52 | 6.06 | 9.74 | 1.98 |
| Mahasamund | 42.02 | 5.79 | 37.85 | 5.18 | 19.35 | 4.07 |
| Mahbubnagar | 32.99 | 5.71 | 31.00 | 5.86 | 17.50 | 3.56 |
| Mahe | 30.14 | 4.95 | 13.83 | 1.99 | 8.14 | 1.40 |
| Mahendragarh | 25.24 | 5.64 | 24.36 | 3.95 | 17.39 | 4.18 |
| Mahesana | 40.35 | 5.19 | 41.82 | 5.31 | 23.92 | 6.31 |
| Mahoba | 43.44 | 6.49 | 45.17 | 5.31 | 21.68 | 4.01 |
| Mahrajganj | 52.91 | 5.15 | 36.85 | 5.33 | 11.99 | 2.50 |
| Mainpuri | 46.11 | 7.34 | 33.19 | 6.96 | 11.00 | 2.13 |
| Malappuram | 23.45 | 4.29 | 16.23 | 2.59 | 20.22 | 5.04 |
| Maldah | 36.70 | 6.01 | 36.57 | 4.80 | 22.56 | 6.85 |
| Malkangiri | 43.71 | 7.01 | 49.18 | 6.94 | 30.49 | 5.82 |
| Mamit | 29.89 | 6.37 | 16.19 | 4.33 | 7.94 | 1.74 |
| Mandi | 24.11 | 3.96 | 16.50 | 3.51 | 12.67 | 3.82 |
| Mandla | 36.74 | 5.60 | 48.28 | 5.56 | 32.40 | 4.79 |
| Mandsaur | 33.49 | 4.03 | 32.07 | 4.43 | 21.51 | 3.61 |
| Mandya | 23.55 | 3.16 | 22.02 | 2.73 | 22.03 | 3.74 |
| Mansa | 27.27 | 4.58 | 23.57 | 3.83 | 16.12 | 4.26 |
| Mathura | 40.37 | 6.43 | 26.72 | 4.58 | 11.70 | 3.01 |
| Mau | 41.16 | 7.03 | 35.09 | 5.32 | 19.39 | 4.83 |
| Mayurbhanj | 40.71 | 5.91 | 40.91 | 5.79 | 16.23 | 3.71 |
| Medak | 31.88 | 3.94 | 35.66 | 5.21 | 20.10 | 3.59 |
| Meerut | 35.05 | 6.85 | 33.91 | 6.31 | 18.31 | 3.95 |
| Mewat | 50.51 | 8.28 | 38.88 | 7.49 | 16.85 | 4.10 |
| Mirzapur | 49.87 | 6.50 | 46.84 | 6.60 | 19.00 | 4.74 |
| Moga | 27.32 | 4.86 | 22.78 | 4.02 | 17.77 | 3.78 |
| Mokokchung | 22.59 | 3.19 | 10.50 | 1.47 | 5.71 | 0.51 |
| Mon | 35.71 | 6.71 | 22.63 | 5.78 | 19.11 | 5.94 |
| Moradabad | 43.26 | 6.90 | 40.47 | 6.19 | 15.06 | 3.28 |
| Morena | 45.15 | 8.40 | 51.10 | 6.76 | 30.36 | 6.45 |
| Morigaon | 34.74 | 7.92 | 24.52 | 5.58 | 10.11 | 2.32 |
| Muktsar | 28.72 | 5.49 | 20.63 | 4.20 | 14.82 | 3.57 |
| Mumbai | 29.64 | 4.09 | 28.38 | 4.50 | 24.27 | 3.32 |
| Mumbai suburban | 25.31 | 3.10 | 29.25 | 4.05 | 22.40 | 4.24 |
| Munger | 46.62 | 6.07 | 44.34 | 6.30 | 19.66 | 5.37 |
| Murshidabad | 39.50 | 6.66 | 33.42 | 7.02 | 15.84 | 3.18 |
| Muzaffarnagar | 40.58 | 6.42 | 37.12 | 5.83 | 19.24 | 4.76 |
| Muzaffarpur | 46.87 | 6.46 | 41.51 | 6.65 | 17.23 | 3.66 |
| Mysore | 27.68 | 3.40 | 26.73 | 3.54 | 17.90 | 3.44 |
| Nabarangapur | 43.15 | 6.29 | 47.84 | 6.07 | 32.80 | 6.00 |
| Nadia | 24.35 | 3.34 | 20.88 | 3.18 | 11.90 | 1.90 |
| Nagaon | 36.28 | 6.32 | 29.50 | 5.42 | 12.85 | 2.65 |
| Nagapattinam | 23.17 | 3.58 | 21.95 | 3.08 | 17.44 | 4.06 |
| Nagaur | 38.39 | 5.84 | 31.09 | 5.81 | 18.18 | 4.79 |
| Nagpur | 29.90 | 4.39 | 31.15 | 4.84 | 23.12 | 4.13 |
| Nainital | 30.05 | 5.25 | 17.02 | 2.87 | 9.72 | 2.30 |
| Nalanda | 52.93 | 7.15 | 47.37 | 7.52 | 22.37 | 5.60 |
| Nalbari | 27.54 | 4.14 | 20.62 | 3.56 | 14.54 | 3.63 |
| Nalgonda | 28.08 | 4.92 | 31.06 | 4.80 | 21.03 | 4.66 |
| Namakkal | 26.04 | 4.91 | 19.30 | 3.11 | 14.62 | 2.58 |
| Nanded | 38.78 | 7.34 | 33.78 | 6.26 | 20.13 | 4.40 |
| Nandurbar | 44.04 | 6.42 | 52.31 | 6.15 | 34.77 | 5.44 |
| Narayanpur | 47.23 | 9.86 | 48.13 | 8.13 | 29.32 | 11.25 |
| Narmada | 46.69 | 6.86 | 53.14 | 6.97 | 34.31 | 6.40 |
| Narsimhapur | 38.63 | 4.78 | 35.23 | 4.68 | 21.88 | 3.74 |
| Nashik | 40.62 | 6.47 | 43.01 | 6.27 | 29.76 | 6.30 |
| Navsari | 36.46 | 5.50 | 34.65 | 5.35 | 23.19 | 4.84 |
| Nawada | 48.87 | 7.72 | 45.90 | 6.12 | 20.66 | 4.33 |
| Nayagarh | 27.84 | 5.33 | 25.72 | 4.54 | 16.59 | 3.25 |
| Neemuch | 36.95 | 5.53 | 39.39 | 6.31 | 22.98 | 5.19 |
| New delhi | 25.15 | 3.55 | 25.72 | 3.65 | 20.50 | 4.03 |
| Nicobars | 24.90 | 4.15 | 15.46 | 2.54 | 8.13 | 1.15 |
| Nizamabad | 33.16 | 4.99 | 35.01 | 4.57 | 20.62 | 3.74 |
| North | 27.58 | 3.87 | 25.81 | 3.36 | 15.11 | 2.51 |
| North district | 23.10 | 3.17 | 16.74 | 2.74 | 18.84 | 4.10 |
| North & middle andaman | 30.91 | 5.75 | 29.54 | 5.18 | 26.49 | 6.39 |
| North east | 27.42 | 3.89 | 22.59 | 2.74 | 12.26 | 2.13 |
| North goa | 22.30 | 3.39 | 23.16 | 3.43 | 15.63 | 2.88 |
| North tripura | 27.39 | 5.42 | 28.59 | 5.63 | 14.05 | 3.10 |
| North twenty four parganas | 24.46 | 3.29 | 19.38 | 2.85 | 13.93 | 2.54 |
| North west | 34.19 | 3.87 | 28.71 | 3.66 | 16.65 | 2.32 |
| Nuapada | 36.48 | 5.84 | 39.83 | 6.95 | 25.05 | 5.05 |
| Osmanabad | 40.24 | 5.01 | 42.09 | 4.46 | 20.38 | 4.17 |
| Pakur | 50.48 | 7.75 | 46.82 | 6.61 | 24.68 | 4.78 |
| Palakkad | 19.52 | 3.38 | 17.77 | 3.85 | 11.34 | 2.62 |
| Palamu | 45.34 | 6.22 | 44.55 | 6.24 | 23.53 | 5.92 |
| Pali | 40.58 | 6.30 | 38.16 | 6.70 | 20.46 | 5.01 |
| Palwal | 33.53 | 7.13 | 26.89 | 4.58 | 19.98 | 5.49 |
| Panchkula | 25.76 | 4.39 | 28.22 | 4.92 | 32.14 | 5.73 |
| Panchmahal | 40.20 | 5.48 | 42.06 | 5.92 | 35.00 | 6.52 |
| Panipat | 41.20 | 6.37 | 38.31 | 5.74 | 24.12 | 5.09 |
| Panna | 41.84 | 5.85 | 40.84 | 5.43 | 23.39 | 4.55 |
| Papumpare | 25.71 | 3.94 | 12.17 | 1.90 | 10.20 | 1.56 |
| Parbhani | 42.94 | 6.90 | 39.61 | 4.87 | 18.77 | 3.59 |
| Paschim medinipur | 29.31 | 4.26 | 39.33 | 4.85 | 25.91 | 5.06 |
| Pashchim champaran | 44.33 | 6.46 | 39.04 | 6.51 | 19.67 | 4.49 |
| Pashchimi singhbhum | 57.65 | 6.16 | 65.24 | 4.96 | 36.33 | 6.71 |
| Patan | 37.45 | 4.82 | 38.51 | 5.45 | 23.62 | 4.82 |
| Pathanamthitta | 15.62 | 1.96 | 13.00 | 1.61 | 14.32 | 2.22 |
| Patiala | 21.34 | 3.73 | 15.23 | 1.87 | 11.88 | 2.43 |
| Patna | 43.18 | 7.67 | 43.51 | 6.65 | 27.28 | 7.27 |
| Perambalur | 25.29 | 4.14 | 22.85 | 3.75 | 17.82 | 3.13 |
| Peren | 29.24 | 6.19 | 11.16 | 2.82 | 5.38 | 1.00 |
| Phek | 25.41 | 5.38 | 12.33 | 2.31 | 6.83 | 1.27 |
| Pilibhit | 50.46 | 7.82 | 43.20 | 5.84 | 20.50 | 3.12 |
| Pithoragarh | 29.21 | 5.74 | 17.37 | 2.67 | 20.42 | 5.20 |
| Porbandar | 24.97 | 4.20 | 28.63 | 3.84 | 24.16 | 5.52 |
| Prakasam | 27.00 | 4.81 | 29.85 | 4.23 | 15.06 | 2.67 |
| Pratapgarh | 44.25 | 6.89 | 52.52 | 6.85 | 35.84 | 7.12 |
| Pratapgarh | 41.43 | 6.04 | 42.25 | 6.22 | 22.63 | 4.55 |
| Puducherry | 23.31 | 3.88 | 19.55 | 2.58 | 20.64 | 4.30 |
| Pudukkottai | 26.40 | 4.20 | 24.70 | 3.72 | 19.63 | 3.92 |
| Pulwama | 22.90 | 3.64 | 12.05 | 2.22 | 9.76 | 2.36 |
| Punch | 26.70 | 6.78 | 20.15 | 5.69 | 15.67 | 4.27 |
| Pune | 24.31 | 4.01 | 26.50 | 3.37 | 21.63 | 4.19 |
| Purba champaran | 47.09 | 6.96 | 40.99 | 6.54 | 17.33 | 3.69 |
| Purba medinipur | 29.12 | 4.52 | 32.34 | 4.41 | 22.77 | 4.35 |
| Purbi singhbhum | 41.15 | 5.51 | 50.85 | 5.83 | 39.90 | 6.81 |
| Puri | 18.68 | 3.43 | 19.13 | 3.40 | 12.27 | 2.71 |
| Purnia | 52.73 | 6.06 | 47.75 | 7.30 | 20.68 | 4.92 |
| Puruliya | 40.91 | 6.77 | 53.95 | 6.00 | 32.39 | 5.86 |
| Rae bareli | 38.00 | 6.97 | 40.89 | 6.42 | 28.78 | 7.73 |
| Raichur | 36.92 | 6.68 | 40.93 | 6.00 | 33.49 | 7.71 |
| Raigarh | 38.17 | 5.71 | 36.86 | 4.48 | 18.62 | 3.54 |
| Raigarh | 33.16 | 4.41 | 37.88 | 4.72 | 23.98 | 4.19 |
| Raipur | 37.49 | 5.99 | 35.52 | 5.51 | 17.07 | 3.78 |
| Raisen | 45.07 | 6.97 | 44.21 | 7.21 | 23.78 | 4.60 |
| Rajgarh | 39.89 | 7.46 | 47.33 | 5.84 | 31.29 | 6.25 |
| Rajkot | 31.05 | 4.19 | 30.81 | 3.78 | 23.83 | 3.78 |
| Rajnandgaon | 48.28 | 9.19 | 36.62 | 7.73 | 17.08 | 5.30 |
| Rajouri | 34.29 | 6.68 | 20.32 | 3.49 | 10.34 | 2.36 |
| Rajsamand | 38.10 | 5.95 | 38.75 | 7.12 | 27.77 | 6.80 |
| Ramanagara | 23.83 | 4.14 | 23.34 | 3.82 | 19.69 | 3.48 |
| Ramanathapuram | 22.61 | 3.01 | 21.59 | 2.78 | 16.00 | 2.80 |
| Ramban | 31.96 | 6.22 | 16.84 | 2.92 | 14.05 | 4.07 |
| Ramgarh | 37.43 | 6.33 | 46.05 | 6.01 | 30.25 | 6.24 |
| Rampur | 44.44 | 6.00 | 43.21 | 6.28 | 19.79 | 3.82 |
| Ranchi | 39.78 | 5.74 | 42.53 | 5.62 | 26.27 | 4.57 |
| Rangareddy | 26.61 | 3.99 | 25.83 | 3.84 | 15.74 | 2.69 |
| Ratlam | 44.00 | 7.43 | 42.17 | 6.02 | 21.46 | 4.69 |
| Ratnagiri | 31.24 | 4.17 | 34.29 | 4.21 | 24.10 | 3.63 |
| Rayagada | 41.80 | 5.95 | 40.84 | 5.65 | 22.17 | 4.12 |
| Reasi | 26.55 | 5.22 | 22.84 | 5.59 | 16.07 | 4.27 |
| Rewa | 40.58 | 5.90 | 36.17 | 5.21 | 17.71 | 2.89 |
| Rewari | 28.29 | 4.95 | 23.12 | 3.45 | 17.80 | 3.84 |
| Ribhoi | 49.51 | 5.63 | 29.71 | 5.13 | 9.77 | 2.48 |
| Rohtak | 34.62 | 6.01 | 23.43 | 3.56 | 13.71 | 3.90 |
| Rohtas | 48.94 | 7.01 | 45.87 | 7.06 | 19.81 | 4.77 |
| Rudraprayag | 29.62 | 4.34 | 25.85 | 4.65 | 18.21 | 3.66 |
| Rupnagar | 19.08 | 2.44 | 19.55 | 3.15 | 13.16 | 2.38 |
| Sabarkantha | 48.68 | 6.36 | 45.47 | 5.89 | 23.37 | 3.82 |
| Sagar | 39.96 | 5.69 | 30.42 | 4.52 | 17.03 | 3.34 |
| Saharanpur | 35.95 | 6.34 | 34.90 | 5.81 | 16.57 | 4.05 |
| Saharsa | 42.81 | 6.95 | 43.69 | 7.48 | 24.57 | 7.64 |
| Sahibganj | 49.33 | 8.36 | 48.94 | 6.41 | 24.57 | 5.67 |
| Sahibzada ajit singh nagar | 22.25 | 4.25 | 22.13 | 3.77 | 10.66 | 2.14 |
| Saiha | 35.13 | 6.98 | 15.69 | 3.20 | 10.07 | 2.67 |
| Salem | 25.93 | 4.73 | 21.99 | 3.41 | 20.87 | 4.80 |
| Samastipur | 48.83 | 8.23 | 40.84 | 7.09 | 17.82 | 3.92 |
| Samba | 21.23 | 3.57 | 10.22 | 1.33 | 6.13 | 1.16 |
| Sambalpur | 38.10 | 5.91 | 43.26 | 5.59 | 27.19 | 4.79 |
| Sangli | 25.89 | 4.94 | 26.10 | 4.32 | 16.56 | 2.26 |
| Sangrur | 23.23 | 3.99 | 23.11 | 3.31 | 14.78 | 2.45 |
| Sant kabir nagar | 49.96 | 6.82 | 36.32 | 5.98 | 10.63 | 1.92 |
| Sant ravidas nagar (bhadohi) | 50.90 | 6.32 | 48.26 | 6.08 | 21.03 | 4.64 |
| Saraikela kharsawan | 44.86 | 5.91 | 53.70 | 5.11 | 24.00 | 5.01 |
| Saran | 45.88 | 6.45 | 39.76 | 6.62 | 17.46 | 4.10 |
| Satara | 24.16 | 2.82 | 28.12 | 3.31 | 22.70 | 4.26 |
| Satna | 40.70 | 6.39 | 38.68 | 5.72 | 26.14 | 4.33 |
| Sawai madhopur | 38.83 | 6.03 | 34.30 | 5.25 | 16.84 | 3.37 |
| Sehore | 33.13 | 6.14 | 39.34 | 6.98 | 25.77 | 5.52 |
| Senapati (excluding 3 sub-divisions) | 33.83 | 6.51 | 13.49 | 3.01 | 7.16 | 1.98 |
| Seoni | 34.42 | 4.97 | 44.86 | 6.04 | 32.12 | 5.69 |
| Serchhip | 23.48 | 4.78 | 11.29 | 1.72 | 8.88 | 2.29 |
| Shahdol | 37.69 | 5.80 | 41.05 | 4.97 | 25.73 | 4.44 |
| Shahid bhagat singh nagar | 25.13 | 4.68 | 19.00 | 3.97 | 16.86 | 4.85 |
| Shahjahanpur | 48.75 | 6.70 | 53.38 | 6.75 | 22.78 | 5.33 |
| Shajapur | 47.70 | 6.41 | 46.11 | 7.36 | 27.45 | 5.80 |
| Sheikhpura | 46.98 | 8.42 | 50.11 | 8.06 | 26.10 | 7.26 |
| Sheohar | 52.69 | 6.41 | 42.25 | 7.47 | 13.82 | 3.24 |
| Sheopur | 50.04 | 8.95 | 54.27 | 5.81 | 27.03 | 5.75 |
| Shimla | 25.69 | 5.67 | 21.97 | 4.43 | 13.03 | 2.81 |
| Shimoga | 33.75 | 3.87 | 29.69 | 3.57 | 14.70 | 2.01 |
| Shivpuri | 46.72 | 7.87 | 48.26 | 6.39 | 24.99 | 4.26 |
| Shrawasti | 61.46 | 5.30 | 38.86 | 5.63 | 10.60 | 2.65 |
| Shupiyan | 23.70 | 4.98 | 12.36 | 2.11 | 7.94 | 2.01 |
| Siddharth nagar | 56.76 | 5.82 | 42.72 | 6.68 | 13.42 | 3.32 |
| Sidhi | 47.61 | 6.24 | 44.05 | 6.36 | 24.54 | 4.52 |
| Sikar | 28.78 | 5.43 | 20.91 | 3.54 | 11.97 | 2.69 |
| Simdega | 41.05 | 5.27 | 49.51 | 6.93 | 36.25 | 8.17 |
| Sindhudurg | 27.88 | 3.36 | 27.64 | 2.86 | 20.02 | 2.66 |
| Singrauli | 33.70 | 5.83 | 36.75 | 7.00 | 31.88 | 6.44 |
| Sirmaur | 23.19 | 3.78 | 24.42 | 3.61 | 18.51 | 3.64 |
| Sirohi | 40.36 | 7.24 | 48.36 | 6.86 | 34.89 | 7.04 |
| Sirsa | 33.63 | 6.17 | 29.14 | 6.83 | 21.33 | 4.93 |
| Sitamarhi | 56.65 | 6.98 | 48.16 | 5.49 | 15.82 | 2.46 |
| Sitapur | 54.55 | 7.53 | 47.17 | 6.66 | 13.33 | 3.09 |
| Sivaganga | 20.40 | 3.47 | 21.17 | 3.27 | 17.60 | 3.39 |
| Sivasagar | 34.95 | 5.67 | 22.40 | 4.73 | 8.92 | 1.35 |
| Siwan | 38.22 | 6.66 | 32.49 | 6.18 | 14.65 | 2.66 |
| Solan | 27.56 | 3.81 | 28.92 | 4.67 | 16.31 | 3.36 |
| Solapur | 27.75 | 3.65 | 31.87 | 4.99 | 22.27 | 4.13 |
| Sonbhadra | 44.86 | 6.90 | 44.78 | 6.43 | 21.21 | 4.99 |
| Sonipat | 40.20 | 7.35 | 30.12 | 4.97 | 20.96 | 5.16 |
| Sonitpur | 28.72 | 4.79 | 25.44 | 4.51 | 18.52 | 6.01 |
| South | 28.25 | 4.08 | 26.75 | 3.54 | 20.87 | 4.27 |
| South andaman | 21.68 | 2.87 | 17.03 | 2.30 | 13.53 | 3.04 |
| South district | 31.25 | 5.27 | 17.45 | 2.35 | 16.20 | 3.68 |
| South garo hills | 20.89 | 3.65 | 31.34 | 4.34 | 32.61 | 6.27 |
| South goa | 17.58 | 2.44 | 20.95 | 3.08 | 22.98 | 6.18 |
| South tripura | 23.32 | 2.86 | 24.94 | 3.42 | 19.80 | 3.81 |
| South twenty four parganas | 27.84 | 5.59 | 28.18 | 4.34 | 19.05 | 4.32 |
| South west | 28.60 | 3.93 | 20.65 | 2.80 | 14.36 | 2.55 |
| Sri potti sriramulu nellore | 29.24 | 4.25 | 28.61 | 5.64 | 16.47 | 3.47 |
| Srikakulam | 28.18 | 3.91 | 28.95 | 4.60 | 14.67 | 2.43 |
| Srinagar | 27.83 | 5.62 | 20.17 | 4.19 | 21.99 | 6.18 |
| Subarnapur | 43.42 | 5.49 | 41.71 | 5.28 | 21.15 | 4.19 |
| Sultanpur | 44.92 | 6.89 | 37.89 | 5.19 | 17.72 | 3.67 |
| Sundargarh | 35.41 | 5.37 | 40.59 | 5.29 | 28.31 | 5.49 |
| Supaul | 48.60 | 7.16 | 43.65 | 7.02 | 20.47 | 4.37 |
| Surat | 29.14 | 4.98 | 34.76 | 4.27 | 25.76 | 4.29 |
| Surendranagar | 44.39 | 5.16 | 45.18 | 4.79 | 26.26 | 4.76 |
| Surguja | 31.51 | 4.70 | 34.71 | 5.14 | 21.82 | 4.30 |
| Tamenglong | 35.78 | 7.92 | 14.67 | 3.16 | 7.49 | 1.47 |
| Tapi | 35.77 | 4.31 | 42.31 | 6.40 | 34.40 | 5.38 |
| Tarn taran | 23.33 | 3.79 | 14.67 | 2.37 | 9.42 | 1.97 |
| Tawang | 19.61 | 1.98 | 10.57 | 1.15 | 17.09 | 2.69 |
| Tehri garhwal | 29.15 | 5.66 | 39.78 | 6.57 | 44.82 | 8.39 |
| Thane | 38.23 | 6.10 | 40.06 | 5.86 | 26.91 | 5.16 |
| Thanjavur | 25.94 | 4.15 | 23.96 | 4.21 | 19.90 | 3.23 |
| The dangs | 44.52 | 5.84 | 56.95 | 5.89 | 41.36 | 6.57 |
| The nilgiris | 30.30 | 4.17 | 28.17 | 4.28 | 27.27 | 6.32 |
| Theni | 27.89 | 5.20 | 24.55 | 4.05 | 14.92 | 2.58 |
| Thiruvallur | 30.36 | 4.08 | 28.73 | 3.88 | 23.09 | 4.53 |
| Thiruvananthapuram | 18.73 | 2.91 | 19.70 | 2.60 | 13.76 | 2.53 |
| Thiruvarur | 26.32 | 4.63 | 27.73 | 3.79 | 20.81 | 3.92 |
| Thoothukkudi | 21.51 | 3.22 | 19.06 | 2.92 | 13.17 | 2.62 |
| Thoubal | 27.66 | 5.57 | 15.48 | 3.36 | 6.86 | 1.23 |
| Thrissur | 19.20 | 2.23 | 12.97 | 1.37 | 13.29 | 2.01 |
| Tikamgarh | 49.02 | 6.78 | 43.72 | 6.23 | 19.55 | 3.49 |
| Tinsukia | 35.60 | 6.43 | 31.34 | 5.79 | 13.30 | 2.72 |
| Tirap | 35.53 | 5.34 | 28.88 | 5.35 | 21.50 | 5.93 |
| Tiruchirappalli | 27.97 | 4.84 | 26.98 | 4.05 | 19.13 | 2.97 |
| Tirunelveli | 28.70 | 3.91 | 22.46 | 3.00 | 13.44 | 1.94 |
| Tiruppur | 27.72 | 4.57 | 25.85 | 3.57 | 22.55 | 4.44 |
| Tiruvannamalai | 23.98 | 3.21 | 30.99 | 5.12 | 30.59 | 8.98 |
| Tonk | 31.42 | 5.39 | 34.87 | 5.16 | 21.69 | 5.11 |
| Tuensang | 32.61 | 5.35 | 18.66 | 3.78 | 11.26 | 3.14 |
| Tumkur | 28.12 | 3.69 | 25.83 | 3.69 | 24.91 | 4.68 |
| Udaipur | 44.74 | 7.95 | 47.98 | 7.81 | 28.35 | 6.42 |
| Udalguri | 36.77 | 5.98 | 30.31 | 4.86 | 17.06 | 4.49 |
| Udham singh nagar | 35.73 | 6.50 | 26.35 | 4.55 | 11.85 | 2.20 |
| Udhampur | 40.21 | 6.89 | 25.06 | 5.39 | 12.54 | 2.92 |
| Udupi | 24.85 | 2.97 | 24.08 | 3.51 | 20.13 | 2.80 |
| Ujjain | 35.91 | 6.39 | 32.06 | 5.10 | 18.59 | 4.06 |
| Ukhrul | 32.28 | 5.24 | 11.30 | 1.74 | 6.52 | 1.43 |
| Umaria | 40.43 | 6.04 | 45.24 | 5.89 | 25.53 | 5.16 |
| Una | 23.17 | 3.26 | 15.62 | 2.28 | 11.14 | 2.54 |
| Unnao | 45.71 | 6.58 | 34.22 | 5.98 | 12.56 | 2.98 |
| Upper siang | 25.03 | 4.25 | 17.56 | 2.92 | 21.29 | 5.42 |
| Upper subansiri | 28.92 | 4.22 | 12.33 | 1.68 | 12.80 | 2.41 |
| Uttar bastar kanker | 35.95 | 5.55 | 47.28 | 5.60 | 29.07 | 6.43 |
| Uttar dinajpur | 37.65 | 7.83 | 33.16 | 6.53 | 14.08 | 3.41 |
| Uttara Kannada | 37.16 | 4.68 | 30.82 | 3.93 | 19.19 | 3.75 |
| Uttarkashi | 33.14 | 5.12 | 38.62 | 6.25 | 37.99 | 8.25 |
| Vadodara | 41.97 | 5.02 | 39.08 | 5.54 | 17.56 | 3.05 |
| Vaishali | 52.70 | 6.48 | 40.46 | 6.47 | 14.07 | 4.04 |
| Valsad | 40.51 | 7.02 | 41.24 | 5.79 | 29.54 | 6.06 |
| Varanasi | 44.01 | 7.08 | 45.40 | 7.05 | 23.84 | 5.58 |
| Vellore | 28.52 | 4.33 | 30.37 | 3.99 | 25.52 | 5.59 |
| Vidisha | 41.31 | 5.23 | 40.99 | 4.86 | 21.13 | 4.59 |
| Viluppuram | 30.03 | 4.90 | 27.53 | 4.94 | 16.20 | 2.80 |
| Virudhunagar | 29.11 | 4.22 | 25.49 | 3.11 | 16.39 | 2.32 |
| Visakhapatnam | 30.11 | 3.57 | 32.43 | 4.36 | 16.39 | 3.08 |
| Vizianagaram | 35.96 | 5.36 | 33.18 | 5.04 | 16.73 | 3.55 |
| Warangal | 26.21 | 4.29 | 28.83 | 3.98 | 16.38 | 2.66 |
| Wardha | 30.97 | 5.35 | 37.82 | 4.63 | 24.97 | 4.30 |
| Washim | 39.19 | 5.84 | 42.28 | 5.42 | 30.69 | 6.15 |
| Wayanad | 23.98 | 3.84 | 22.60 | 3.94 | 19.77 | 4.83 |
| West | 32.92 | 4.74 | 28.20 | 3.49 | 16.88 | 3.14 |
| West district | 38.01 | 5.46 | 13.61 | 2.73 | 13.00 | 3.08 |
| West garo hills | 29.83 | 5.62 | 24.38 | 5.34 | 19.36 | 4.54 |
| West godavari | 28.79 | 3.76 | 30.29 | 4.02 | 15.10 | 2.51 |
| West kameng | 26.69 | 4.48 | 13.19 | 1.87 | 8.50 | 1.21 |
| West khasi hills | 48.96 | 9.57 | 28.89 | 5.98 | 15.99 | 6.28 |
| West siang | 30.71 | 4.44 | 18.86 | 2.28 | 16.55 | 4.42 |
| West tripura | 19.05 | 2.93 | 19.45 | 2.81 | 14.04 | 2.43 |
| Wokha | 22.25 | 3.15 | 14.19 | 2.35 | 11.96 | 3.01 |
| Y.S.R | 33.25 | 4.79 | 33.04 | 4.56 | 16.47 | 3.36 |
| Yadgir | 52.13 | 6.69 | 48.20 | 6.93 | 30.15 | 7.03 |
| Yamunanagar | 29.46 | 5.77 | 31.18 | 4.47 | 25.98 | 5.78 |
| Yanam | 30.33 | 4.42 | 25.37 | 4.39 | 15.70 | 2.63 |
| Yavatmal | 42.68 | 7.01 | 45.57 | 5.51 | 28.76 | 5.76 |
| Zunheboto | 28.75 | 5.11 | 13.99 | 1.94 | 9.74 | 1.64 |

**Table S3:** Mean Prevalence and Standard Deviation of Child Anthropometric Failures by Parliamentary Constituencies, India, NFHS, 2016

|  | **Stunting** | | **Underweight** | | **Wasting** | |
| --- | --- | --- | --- | --- | --- | --- |
| **Parliamentary Constituencies** | **Mean** | **SD** | **Mean** | **SD** | **Mean** | **SD** |
| Adilabad | 36.20 | 6.39 | 35.82 | 5.22 | 22.36 | 4.52 |
| Agra | 42.27 | 7.24 | 32.95 | 5.24 | 9.54 | 2.44 |
| Ahmadabad (East) | 36.17 | 5.37 | 40.90 | 6.96 | 20.72 | 3.48 |
| Ahmadabad (West) | 29.22 | 3.30 | 29.58 | 3.75 | 29.75 | 8.58 |
| Ahmadnagar | 32.82 | 4.35 | 32.48 | 5.57 | 22.83 | 4.97 |
| Ajmer | 32.29 | 5.56 | 37.40 | 6.72 | 24.26 | 6.36 |
| Akbarpur | 42.10 | 6.80 | 43.58 | 6.54 | 22.10 | 7.00 |
| Akola | 36.07 | 6.84 | 38.09 | 5.97 | 25.15 | 5.66 |
| Alappuzha | 15.60 | 1.69 | 10.63 | 1.53 | 13.27 | 3.03 |
| Alathur | 20.69 | 2.22 | 13.76 | 1.42 | 10.40 | 1.71 |
| Aligarh | 45.44 | 7.55 | 41.60 | 7.54 | 13.30 | 4.22 |
| Alipurduars | 34.34 | 4.71 | 31.76 | 4.27 | 20.48 | 3.74 |
| Allahabad | 43.82 | 6.91 | 38.41 | 7.91 | 18.26 | 5.00 |
| Almora | 28.79 | 5.77 | 21.67 | 3.45 | 16.98 | 3.59 |
| Alwar | 41.11 | 7.68 | 19.63 | 3.99 | 14.00 | 2.80 |
| Ambala | 23.09 | 4.35 | 32.60 | 6.41 | 24.12 | 6.13 |
| Ambedkar Nagar | 42.75 | 6.95 | 48.04 | 6.04 | 20.92 | 4.74 |
| Amethi | 45.26 | 7.68 | 29.96 | 5.65 | 13.65 | 2.66 |
| Amlapuram | 26.27 | 2.86 | 29.57 | 4.37 | 11.73 | 1.29 |
| Amravati | 33.80 | 4.74 | 29.38 | 5.10 | 21.95 | 4.71 |
| Amreli | 38.67 | 5.18 | 33.62 | 5.62 | 26.34 | 4.00 |
| Amritsar | 22.20 | 5.07 | 20.44 | 4.22 | 15.19 | 4.69 |
| Amroha | 41.44 | 7.49 | 32.70 | 5.42 | 14.11 | 2.75 |
| Anakapalli | 31.98 | 4.07 | 30.09 | 4.42 | 13.53 | 3.02 |
| Anand | 44.22 | 6.15 | 38.35 | 5.55 | 24.34 | 4.24 |
| Anandpur Sahib | 21.24 | 3.47 | 15.45 | 2.95 | 11.22 | 3.30 |
| Anantapur | 40.55 | 6.58 | 41.55 | 5.86 | 16.48 | 2.42 |
| Anantnag | 21.45 | 4.35 | 12.70 | 2.81 | 7.32 | 2.01 |
| Andaman & Nicobar Islands | 25.78 | 4.53 | 19.54 | 3.54 | 14.32 | 3.55 |
| Aonla | 50.18 | 8.11 | 39.68 | 5.84 | 17.39 | 3.97 |
| Arakkonam | 27.42 | 4.50 | 26.87 | 4.74 | 22.61 | 5.51 |
| Araku | 29.33 | 4.21 | 33.32 | 5.85 | 20.74 | 3.50 |
| Arambag | 37.49 | 5.11 | 32.62 | 4.16 | 13.25 | 2.97 |
| Arani | 23.83 | 3.60 | 21.02 | 3.50 | 19.26 | 2.72 |
| Araria | 47.49 | 7.34 | 45.06 | 6.74 | 21.68 | 5.49 |
| Arrah | 43.73 | 6.95 | 49.16 | 6.63 | 26.24 | 8.37 |
| Arunachal East | 30.21 | 5.28 | 16.22 | 2.93 | 13.85 | 3.91 |
| Arunachal West | 26.17 | 4.73 | 18.80 | 3.72 | 17.92 | 4.83 |
| Asansol | 29.51 | 5.51 | 33.78 | 6.51 | 26.62 | 4.32 |
| Aska | 29.17 | 6.20 | 17.91 | 2.61 | 13.54 | 2.84 |
| Attingal | 19.82 | 3.35 | 12.81 | 2.28 | 10.64 | 1.75 |
| Aurangabad | 52.13 | 8.00 | 50.90 | 8.24 | 25.19 | 5.33 |
| Aurangabad | 33.83 | 5.89 | 37.89 | 5.02 | 22.22 | 5.05 |
| Autonomous District | 30.71 | 6.26 | 20.87 | 4.48 | 12.12 | 4.33 |
| Azamgarh | 40.03 | 7.06 | 35.25 | 6.28 | 13.24 | 3.42 |
| Bagalkot | 45.68 | 5.71 | 17.87 | 3.60 | 14.48 | 2.10 |
| Baghpat | 34.39 | 6.22 | 34.70 | 6.50 | 18.62 | 5.23 |
| Baharampur | 35.98 | 6.59 | 27.68 | 5.37 | 13.65 | 2.66 |
| Bahraich | 63.25 | 5.58 | 52.19 | 6.96 | 22.64 | 5.29 |
| Balaghat | 33.23 | 4.83 | 38.41 | 6.25 | 26.95 | 5.41 |
| Baleshwar | 34.12 | 5.11 | 36.87 | 6.04 | 17.19 | 3.59 |
| Ballia | 42.89 | 7.12 | 49.38 | 6.86 | 26.40 | 6.20 |
| Balurghat | 33.83 | 6.04 | 27.57 | 4.02 | 16.79 | 2.85 |
| Banas Kantha | 39.56 | 6.85 | 42.57 | 7.19 | 26.55 | 6.37 |
| Banda | 48.53 | 7.59 | 39.99 | 6.13 | 21.10 | 4.17 |
| Bangalore Central | 30.08 | 3.88 | 43.62 | 5.57 | 26.94 | 4.84 |
| Bangalore North | 34.89 | 5.20 | 23.96 | 2.64 | 19.05 | 3.22 |
| Bangalore Rural | 23.60 | 4.06 | 41.60 | 6.02 | 32.17 | 5.96 |
| Bangalore South | 28.60 | 3.15 | 30.67 | 4.99 | 22.39 | 4.06 |
| Bangaon | 26.85 | 2.62 | 22.97 | 3.68 | 13.94 | 1.78 |
| Banka | 48.15 | 7.70 | 46.47 | 6.50 | 25.18 | 5.05 |
| Bankura | 33.22 | 5.77 | 35.02 | 6.87 | 21.41 | 5.85 |
| Bansgaon | 39.39 | 7.47 | 30.17 | 3.60 | 14.75 | 2.60 |
| Banswara | 48.28 | 6.73 | 25.92 | 4.63 | 14.73 | 3.10 |
| Bapatla | 24.13 | 2.96 | 28.99 | 4.40 | 14.58 | 2.47 |
| Barabanki | 50.07 | 7.73 | 42.75 | 5.70 | 17.91 | 4.66 |
| Barakpur | 29.54 | 3.87 | 24.09 | 3.17 | 15.49 | 1.57 |
| Baramati | 27.23 | 4.52 | 28.67 | 2.19 | 20.20 | 4.43 |
| Baramula | 26.54 | 5.45 | 20.50 | 4.64 | 14.42 | 4.10 |
| Barasat | 25.58 | 2.04 | 26.15 | 4.18 | 17.92 | 3.76 |
| Barddhaman - Durgapur | 32.89 | 6.29 | 30.06 | 4.24 | 20.19 | 3.38 |
| Barddhaman Purba | 28.05 | 4.07 | 29.66 | 4.61 | 21.51 | 4.51 |
| Bardoli | 36.92 | 4.77 | 43.44 | 6.47 | 26.52 | 5.52 |
| Bareilly | 43.00 | 6.22 | 39.16 | 5.07 | 17.66 | 6.47 |
| Barmer | 34.31 | 6.74 | 31.86 | 5.49 | 16.44 | 4.02 |
| Barpeta | 37.02 | 6.52 | 28.59 | 5.09 | 17.52 | 4.62 |
| Basirhat | 28.16 | 5.33 | 25.57 | 4.08 | 17.83 | 4.43 |
| Bastar | 45.49 | 8.27 | 48.04 | 7.00 | 27.80 | 7.63 |
| Basti | 48.53 | 7.16 | 34.11 | 6.07 | 17.02 | 4.14 |
| Bathinda | 27.17 | 5.22 | 15.73 | 2.57 | 12.24 | 3.11 |
| Beed | 35.37 | 5.67 | 36.53 | 5.35 | 27.91 | 5.30 |
| Begusarai | 44.64 | 7.15 | 38.47 | 7.98 | 18.40 | 5.25 |
| Belgaum | 32.95 | 4.76 | 27.05 | 3.28 | 25.35 | 4.15 |
| Bellary | 46.33 | 6.02 | 31.48 | 5.11 | 29.61 | 5.11 |
| Berhampur. | 31.59 | 4.98 | 22.69 | 3.72 | 15.07 | 3.56 |
| Betul | 36.54 | 6.46 | 50.59 | 7.08 | 23.86 | 6.13 |
| Bhadrak | 34.55 | 6.30 | 27.31 | 4.32 | 14.18 | 3.08 |
| Bhagalpur | 46.88 | 9.16 | 41.60 | 7.67 | 22.11 | 7.25 |
| Bhandara - Gondiya | 34.02 | 5.08 | 34.13 | 5.15 | 23.29 | 4.65 |
| Bharatpur | 43.94 | 7.86 | 33.77 | 5.33 | 18.94 | 4.80 |
| Bharuch | 43.62 | 6.30 | 44.60 | 7.37 | 27.37 | 6.40 |
| Bhavnagar | 45.24 | 5.27 | 19.44 | 3.34 | 11.88 | 2.82 |
| Bhilwara | 33.43 | 4.75 | 45.25 | 7.39 | 29.85 | 7.84 |
| Bhind | 46.93 | 6.82 | 41.95 | 5.32 | 30.57 | 5.28 |
| Bhiwani - Mahendragarh | 28.90 | 6.76 | 33.40 | 6.48 | 17.80 | 4.78 |
| Bhongir | 25.67 | 3.80 | 28.67 | 4.54 | 19.05 | 4.09 |
| Bhopal | 41.94 | 6.97 | 41.99 | 7.08 | 25.31 | 5.09 |
| Bhubaneswar | 21.30 | 3.27 | 20.60 | 3.58 | 11.84 | 2.04 |
| Bidar | 44.94 | 6.35 | 32.14 | 4.18 | 20.58 | 4.34 |
| Bijapur | 41.75 | 6.73 | 33.66 | 5.17 | 26.99 | 6.49 |
| Bijnor | 37.85 | 7.34 | 35.64 | 6.30 | 13.90 | 3.47 |
| Bikaner | 32.47 | 6.05 | 26.65 | 4.43 | 21.24 | 5.69 |
| Bilaspur | 35.22 | 8.12 | 33.88 | 6.63 | 25.86 | 7.64 |
| Birbhum | 38.82 | 6.16 | 42.53 | 5.25 | 29.33 | 6.48 |
| Bishnupur | 31.87 | 4.90 | 40.93 | 6.37 | 28.61 | 6.36 |
| Biwandi | 42.93 | 7.48 | 45.67 | 5.87 | 27.36 | 5.18 |
| Bolangir | 42.28 | 6.11 | 30.91 | 4.98 | 19.31 | 3.56 |
| Bolpur | 35.80 | 5.61 | 32.81 | 6.93 | 16.96 | 2.95 |
| Budaun | 53.38 | 6.85 | 45.46 | 8.10 | 19.25 | 4.81 |
| Bulandshahr | 44.32 | 8.09 | 42.24 | 6.18 | 19.67 | 3.44 |
| Buldana | 40.64 | 6.45 | 40.25 | 5.94 | 21.47 | 4.42 |
| Buxar | 45.80 | 8.80 | 41.08 | 7.07 | 18.34 | 4.73 |
| Chalakudy | 17.41 | 3.18 | 15.04 | 1.89 | 20.65 | 3.68 |
| Chamrajnagar | 31.58 | 3.69 | 21.75 | 2.91 | 20.73 | 3.78 |
| Chandauli | 44.84 | 7.94 | 33.54 | 5.88 | 18.20 | 5.06 |
| Chandigarh | 30.96 | 4.45 | 24.92 | 4.51 | 10.99 | 2.50 |
| Chandni Chowk | 29.07 | 3.45 | 25.94 | 3.06 | 11.27 | 2.11 |
| Chandrapur | 30.50 | 4.49 | 40.04 | 5.00 | 28.75 | 5.24 |
| Chatra | 47.72 | 6.95 | 42.58 | 5.84 | 25.68 | 4.49 |
| Chennai Central | 26.25 | 5.10 | 22.78 | 4.37 | 18.79 | 3.60 |
| Chennai North | 31.72 | 6.09 | 24.83 | 3.68 | 19.79 | 3.44 |
| Chennai South | 27.57 | 5.22 | 26.30 | 3.92 | 19.84 | 3.99 |
| Chevella | 32.57 | 4.64 | 35.53 | 4.88 | 21.96 | 3.72 |
| Chhindwara | 34.89 | 6.23 | 42.09 | 5.97 | 26.53 | 5.52 |
| Chhota Udaipur | 44.89 | 6.43 | 27.16 | 3.68 | 25.11 | 5.45 |
| Chidambaram | 28.76 | 4.56 | 25.88 | 4.22 | 23.87 | 5.84 |
| Chikkaballapura | 33.99 | 4.65 | 25.66 | 3.53 | 20.89 | 3.56 |
| Chikkodi | 34.83 | 6.51 | 46.95 | 6.50 | 35.13 | 8.07 |
| Chitradurga | 29.80 | 4.76 | 40.92 | 6.45 | 22.82 | 5.60 |
| Chittaurgarh | 40.95 | 6.50 | 28.46 | 4.91 | 16.36 | 3.81 |
| Chittoor | 32.02 | 6.77 | 30.94 | 6.21 | 17.21 | 4.05 |
| Churu | 30.77 | 5.41 | 21.00 | 3.59 | 16.83 | 3.74 |
| Coimbatore | 24.63 | 4.31 | 15.17 | 2.35 | 11.19 | 2.59 |
| Cuddalore | 29.45 | 5.72 | 25.56 | 4.61 | 16.54 | 3.09 |
| Cuttack | 19.53 | 2.93 | 23.92 | 4.16 | 12.20 | 2.34 |
| Dadra & Nagar Haveli | 40.98 | 6.89 | 37.12 | 5.82 | 23.00 | 6.18 |
| Dakshina Kannada | 25.26 | 3.31 | 24.58 | 3.81 | 15.33 | 2.68 |
| Daman & Diu | 27.81 | 4.73 | 26.90 | 4.52 | 19.66 | 3.78 |
| Damoh | 42.53 | 6.33 | 41.26 | 5.54 | 26.54 | 5.44 |
| Darbhanga | 47.36 | 7.09 | 40.32 | 6.26 | 15.68 | 3.83 |
| Darjiling | 32.86 | 5.90 | 28.97 | 5.99 | 13.69 | 3.32 |
| Dausa | 34.32 | 5.88 | 25.51 | 4.29 | 18.61 | 4.93 |
| Davanagere | 44.77 | 6.76 | 29.33 | 3.89 | 21.27 | 4.41 |
| Deoria | 44.71 | 7.65 | 44.94 | 5.90 | 28.63 | 5.68 |
| Dewas | 42.31 | 7.64 | 32.78 | 5.30 | 19.11 | 4.45 |
| Dhanbad | 36.27 | 6.42 | 41.41 | 6.56 | 20.65 | 4.95 |
| Dhar | 44.05 | 7.05 | 31.25 | 5.12 | 19.56 | 5.02 |
| Dharmapuri | 24.29 | 3.04 | 25.32 | 4.07 | 19.86 | 3.62 |
| Dharwad | 37.15 | 5.36 | 27.84 | 2.40 | 27.71 | 5.96 |
| Dhaurahra | 56.23 | 8.07 | 35.05 | 5.90 | 14.52 | 3.50 |
| Dhenkanal | 26.48 | 5.23 | 35.78 | 5.11 | 23.02 | 4.13 |
| Dhubri | 43.96 | 7.88 | 38.16 | 6.59 | 20.44 | 6.54 |
| Dhule | 36.00 | 6.87 | 39.81 | 6.10 | 28.64 | 5.72 |
| Diamond Harbour | 27.29 | 5.71 | 22.03 | 2.84 | 18.50 | 2.59 |
| Dibrugarh | 31.15 | 4.98 | 29.36 | 5.39 | 18.20 | 4.33 |
| Dindigul | 27.62 | 5.18 | 22.33 | 3.87 | 19.44 | 3.69 |
| Dindori | 36.91 | 7.23 | 45.80 | 4.97 | 28.87 | 5.48 |
| Dohad | 41.51 | 5.66 | 48.33 | 6.14 | 21.87 | 3.37 |
| Domriaganj | 55.12 | 5.97 | 36.07 | 6.97 | 12.60 | 3.73 |
| Dum Dum | 26.84 | 2.55 | 21.01 | 1.05 | 13.85 | 0.80 |
| Dumka | 43.58 | 6.53 | 46.84 | 6.59 | 24.91 | 7.18 |
| Durg | 33.35 | 6.00 | 33.10 | 6.38 | 18.48 | 3.95 |
| East Delhi | 27.62 | 5.33 | 25.24 | 3.63 | 18.07 | 3.22 |
| Eluru | 26.71 | 3.31 | 28.69 | 3.90 | 15.19 | 3.08 |
| Ernakulam | 15.78 | 2.39 | 19.56 | 3.58 | 17.24 | 4.56 |
| Erode | 32.05 | 5.58 | 14.36 | 2.55 | 14.35 | 3.39 |
| Etah | 49.95 | 5.94 | 42.76 | 6.82 | 14.89 | 3.50 |
| Etawah | 47.91 | 6.44 | 45.11 | 6.36 | 17.92 | 4.47 |
| Faizabad | 50.23 | 6.15 | 37.04 | 6.16 | 12.81 | 2.71 |
| Faridabad | 34.21 | 6.68 | 49.81 | 5.90 | 38.45 | 6.96 |
| Faridkot | 28.95 | 5.59 | 23.74 | 5.83 | 17.60 | 3.97 |
| Farrukhabad | 48.58 | 6.95 | 27.21 | 4.87 | 12.06 | 3.31 |
| Fatehgarh Sahib | 20.97 | 3.88 | 19.07 | 2.96 | 15.49 | 3.09 |
| Fatehpur | 51.22 | 6.62 | 43.80 | 6.69 | 21.68 | 5.36 |
| Fatehpur Sikri | 47.94 | 7.65 | 34.49 | 6.70 | 13.64 | 3.42 |
| Firozabad | 43.60 | 6.50 | 45.30 | 7.89 | 13.23 | 3.38 |
| Firozpur | 27.29 | 5.32 | 19.65 | 3.58 | 14.18 | 2.70 |
| Gandhinagar | 28.13 | 4.71 | 44.71 | 6.77 | 25.56 | 7.45 |
| Ganganagar | 30.64 | 5.29 | 43.05 | 6.91 | 23.33 | 5.12 |
| Garhchiroli - Chimur | 35.95 | 5.70 | 42.04 | 5.45 | 36.36 | 6.68 |
| Garhwal | 28.49 | 4.73 | 37.33 | 6.28 | 36.45 | 7.99 |
| Gautam Buddha Nagar | 31.84 | 6.74 | 26.85 | 5.57 | 12.37 | 3.02 |
| Gaya | 46.75 | 7.75 | 46.67 | 7.16 | 20.71 | 3.78 |
| Ghatal | 29.09 | 4.11 | 32.95 | 4.89 | 23.35 | 5.69 |
| Ghaziabad | 38.20 | 7.32 | 42.31 | 6.50 | 18.93 | 3.89 |
| Ghazipur | 41.04 | 5.21 | 42.14 | 5.72 | 29.80 | 7.31 |
| Ghosi | 39.38 | 6.80 | 34.50 | 6.10 | 17.39 | 3.56 |
| Giridih | 44.86 | 6.33 | 18.36 | 4.00 | 9.73 | 2.66 |
| Godda | 48.75 | 6.48 | 47.44 | 6.59 | 27.46 | 6.41 |
| Gonda | 57.58 | 6.41 | 43.41 | 5.15 | 10.79 | 2.16 |
| Gopalganj | 37.91 | 5.97 | 32.24 | 5.54 | 16.24 | 4.02 |
| Gorakhpur | 43.68 | 6.59 | 35.01 | 5.67 | 24.39 | 5.90 |
| Gulbarga | 50.07 | 7.37 | 35.74 | 5.06 | 26.67 | 4.86 |
| Guna | 42.35 | 7.40 | 40.17 | 5.37 | 28.15 | 5.35 |
| Guntur | 20.66 | 1.95 | 24.46 | 1.99 | 18.80 | 3.15 |
| Gurdaspur | 22.77 | 3.87 | 20.40 | 3.19 | 12.88 | 3.21 |
| Gurgaon | 41.58 | 8.45 | 26.83 | 5.08 | 20.31 | 5.77 |
| Guwahati | 28.50 | 4.96 | 23.98 | 4.73 | 14.20 | 3.38 |
| Gwalior | 43.62 | 7.07 | 40.21 | 6.89 | 28.44 | 5.83 |
| Hajipur | 51.69 | 6.83 | 39.22 | 6.38 | 14.31 | 4.24 |
| Hamirpur | 26.74 | 3.97 | 18.13 | 3.71 | 10.29 | 1.70 |
| Hamirpur | 41.65 | 6.44 | 40.94 | 6.74 | 21.91 | 5.30 |
| Haora | 29.53 | 4.19 | 22.21 | 2.68 | 14.20 | 1.90 |
| Hardoi | 50.64 | 7.34 | 31.46 | 5.05 | 8.85 | 2.36 |
| Hardwar | 35.26 | 6.20 | 21.23 | 3.63 | 18.82 | 4.65 |
| Hassan | 26.90 | 3.36 | 38.89 | 4.05 | 28.72 | 6.50 |
| Hathras | 46.44 | 7.08 | 40.64 | 6.50 | 14.92 | 4.12 |
| Hatkanangle | 24.87 | 4.09 | 28.14 | 5.29 | 22.88 | 5.61 |
| Haveri | 38.18 | 6.46 | 40.67 | 5.93 | 21.17 | 3.43 |
| Hazaribagh | 40.02 | 6.97 | 42.13 | 6.56 | 29.53 | 5.63 |
| Hindupur | 31.92 | 6.70 | 31.20 | 5.69 | 14.44 | 3.99 |
| Hingoli | 37.51 | 5.27 | 34.41 | 4.32 | 23.50 | 4.66 |
| Hisar | 28.06 | 6.07 | 21.52 | 3.93 | 14.56 | 3.69 |
| Hoshangabad | 37.16 | 5.87 | 47.20 | 7.12 | 28.88 | 6.21 |
| Hoshiarpur | 23.51 | 3.62 | 22.40 | 3.41 | 15.78 | 3.34 |
| Hugli | 26.08 | 3.69 | 26.63 | 3.84 | 18.87 | 3.50 |
| Hyderabad | 22.57 | 4.39 | 26.04 | 5.10 | 22.63 | 5.45 |
| Idukki | 16.34 | 1.86 | 14.64 | 1.72 | 14.61 | 1.62 |
| Indore | 36.72 | 6.35 | 48.12 | 6.61 | 27.43 | 6.10 |
| Inner Manipur | 23.60 | 4.73 | 13.54 | 3.03 | 6.84 | 1.57 |
| Jabalpur | 35.08 | 5.51 | 45.06 | 5.89 | 29.23 | 5.71 |
| Jadavpur | 24.09 | 6.68 | 25.11 | 4.40 | 16.39 | 3.34 |
| Jagatsinghpur | 19.24 | 3.66 | 25.53 | 5.40 | 18.59 | 3.50 |
| Jahanabad | 50.84 | 7.69 | 50.08 | 6.90 | 24.64 | 7.84 |
| Jaipur | 31.88 | 5.53 | 46.91 | 7.11 | 27.28 | 6.71 |
| Jaipur Rural | 38.38 | 5.81 | 50.51 | 7.96 | 31.87 | 6.80 |
| Jajapur | 28.77 | 5.61 | 29.61 | 5.18 | 16.24 | 3.16 |
| Jalandhar | 27.79 | 4.52 | 28.32 | 4.99 | 16.80 | 3.91 |
| Jalaun | 43.77 | 4.94 | 36.38 | 5.86 | 18.54 | 4.73 |
| Jalgaon | 32.39 | 4.44 | 33.92 | 4.42 | 30.55 | 5.29 |
| Jalna | 37.68 | 6.62 | 35.95 | 6.22 | 22.15 | 5.48 |
| Jalore | 42.59 | 7.09 | 35.06 | 6.52 | 23.35 | 6.65 |
| Jalpaiguri | 31.92 | 4.97 | 26.22 | 3.99 | 17.73 | 5.25 |
| Jammu | 26.76 | 5.92 | 11.95 | 2.92 | 9.23 | 3.41 |
| Jamnagar | 30.14 | 5.42 | 30.49 | 4.11 | 23.99 | 4.10 |
| Jamshedpur | 40.46 | 5.60 | 60.18 | 6.03 | 31.67 | 6.62 |
| Jamui | 47.31 | 7.95 | 48.68 | 7.75 | 27.50 | 7.11 |
| Jangipur | 40.98 | 6.70 | 36.64 | 7.76 | 21.16 | 4.68 |
| Janjgir-Champa | 34.71 | 6.10 | 35.68 | 4.97 | 21.69 | 4.41 |
| Jaunpur | 46.34 | 6.33 | 39.65 | 5.87 | 14.62 | 2.87 |
| Jaynagar | 36.95 | 7.66 | 34.39 | 6.07 | 22.62 | 8.33 |
| Jhalawar - Baran | 37.53 | 5.14 | 36.80 | 6.95 | 15.85 | 3.61 |
| Jhanjharpur | 53.25 | 7.94 | 46.41 | 5.77 | 18.11 | 5.00 |
| Jhansi | 37.74 | 6.36 | 32.19 | 5.38 | 15.64 | 4.06 |
| Jhargram | 35.16 | 5.31 | 41.79 | 4.94 | 25.77 | 3.98 |
| Jhunjhunun | 31.44 | 6.62 | 30.63 | 6.40 | 22.38 | 5.63 |
| Jodhpur | 40.35 | 7.19 | 34.36 | 6.76 | 17.60 | 3.28 |
| Jorhat | 30.37 | 5.35 | 21.04 | 4.28 | 10.90 | 2.48 |
| Junagadh | 31.11 | 5.47 | 29.72 | 5.49 | 25.28 | 4.35 |
| Kachchh | 38.17 | 6.44 | 36.96 | 6.25 | 30.79 | 8.36 |
| Kadapa | 32.31 | 4.75 | 32.27 | 4.90 | 17.21 | 3.58 |
| Kairana | 42.43 | 7.29 | 32.84 | 5.63 | 15.94 | 4.63 |
| Kaisarganj | 60.90 | 3.68 | 32.75 | 6.95 | 10.22 | 2.17 |
| Kakinada | 35.36 | 3.81 | 31.00 | 4.90 | 14.19 | 2.07 |
| Kalahandi | 36.41 | 6.17 | 41.85 | 5.63 | 22.62 | 5.51 |
| Kaliabor | 34.60 | 5.93 | 25.45 | 4.78 | 12.97 | 3.29 |
| Kallakurichi | 26.20 | 4.91 | 20.58 | 2.60 | 16.21 | 3.00 |
| Kalyan | 29.56 | 2.62 | 26.85 | 2.25 | 19.47 | 3.41 |
| Kancheepuram | 27.89 | 5.00 | 29.92 | 3.78 | 27.26 | 7.23 |
| Kandhamal | 37.15 | 6.44 | 39.53 | 7.05 | 24.12 | 4.71 |
| Kangra | 25.02 | 4.99 | 25.74 | 4.53 | 16.98 | 3.70 |
| Kanker | 34.71 | 5.99 | 45.55 | 6.62 | 29.75 | 7.14 |
| Kannauj | 47.02 | 6.61 | 34.62 | 6.26 | 12.90 | 3.25 |
| Kanniyakumari | 19.69 | 2.90 | 22.74 | 3.06 | 15.97 | 2.46 |
| Kannur | 23.99 | 3.45 | 14.17 | 1.76 | 12.65 | 1.96 |
| Kanpur | 43.00 | 4.09 | 47.96 | 7.85 | 26.46 | 7.53 |
| Kanthi | 31.52 | 4.67 | 33.00 | 4.18 | 21.68 | 4.85 |
| Karakat | 45.73 | 7.24 | 45.69 | 6.89 | 21.98 | 5.10 |
| Karauli - Dhaulpur | 48.73 | 6.67 | 35.14 | 6.91 | 28.12 | 7.53 |
| Karimganj | 38.98 | 7.23 | 33.23 | 5.63 | 17.67 | 4.57 |
| Karimnagar | 24.75 | 4.06 | 25.01 | 2.43 | 18.30 | 3.48 |
| Karnal | 40.40 | 6.86 | 29.32 | 5.52 | 22.75 | 6.04 |
| Karur | 26.04 | 4.32 | 21.76 | 3.31 | 21.63 | 4.62 |
| Kasaragod | 18.60 | 2.70 | 18.67 | 2.75 | 12.58 | 2.02 |
| Katihar | 48.30 | 7.57 | 45.17 | 6.63 | 20.27 | 4.67 |
| Kaushambi | 48.01 | 6.01 | 42.33 | 5.61 | 25.48 | 5.71 |
| Kendrapara | 25.59 | 4.49 | 39.42 | 6.04 | 20.25 | 3.60 |
| Kendujhar | 42.60 | 6.64 | 41.80 | 6.99 | 18.62 | 3.90 |
| Khadoor Sahib | 22.44 | 3.80 | 14.82 | 3.14 | 13.16 | 2.83 |
| Khagaria | 47.52 | 8.44 | 42.63 | 7.83 | 19.62 | 6.38 |
| Khajuraho | 43.20 | 5.85 | 43.92 | 6.07 | 23.07 | 4.88 |
| Khammam | 28.19 | 3.99 | 21.11 | 2.60 | 13.80 | 2.12 |
| Khandwa | 46.36 | 7.51 | 44.44 | 7.26 | 20.27 | 4.86 |
| Khargone | 50.23 | 7.10 | 41.82 | 6.14 | 30.05 | 6.29 |
| Kheda | 41.18 | 6.23 | 39.98 | 5.69 | 27.62 | 4.99 |
| Kheri | 50.03 | 7.38 | 34.38 | 6.24 | 15.48 | 3.42 |
| Khunti | 43.43 | 6.04 | 47.43 | 6.42 | 27.40 | 6.75 |
| Kishanganj | 48.47 | 7.18 | 47.71 | 6.86 | 22.11 | 5.65 |
| Koch Bihar | 28.25 | 5.20 | 26.27 | 3.72 | 17.99 | 3.14 |
| Kodarma | 45.09 | 7.07 | 47.19 | 5.62 | 27.94 | 5.56 |
| Kokrajhar | 33.56 | 6.14 | 23.28 | 4.60 | 10.17 | 2.27 |
| Kolar | 31.55 | 4.74 | 23.08 | 3.75 | 20.14 | 3.69 |
| Kolhapur | 28.23 | 5.02 | 28.66 | 4.89 | 18.39 | 3.29 |
| Kolkata Dakshin | 29.90 | 3.84 | 25.40 | 2.95 | 20.60 | 3.78 |
| Kolkata Uttar | 26.30 | 3.17 | 23.87 | 3.45 | 17.69 | 3.55 |
| Kollam | 16.63 | 2.79 | 14.59 | 2.20 | 16.59 | 3.06 |
| Koppal | 50.21 | 7.95 | 33.41 | 5.14 | 25.10 | 5.54 |
| Koraput | 41.21 | 6.49 | 48.01 | 6.67 | 31.15 | 6.18 |
| Korba | 28.97 | 5.54 | 32.91 | 5.57 | 25.60 | 5.51 |
| Kota | 32.99 | 5.83 | 40.45 | 4.85 | 32.23 | 6.06 |
| Kottayam | 17.70 | 2.46 | 12.48 | 1.61 | 13.41 | 2.77 |
| Kozhikode | 19.47 | 3.40 | 12.85 | 1.67 | 12.15 | 2.42 |
| Krishnagiri | 22.53 | 3.43 | 22.37 | 4.40 | 17.89 | 5.14 |
| Krishnanagar | 24.44 | 4.22 | 21.93 | 3.22 | 14.21 | 2.49 |
| Krukshetra | 32.44 | 6.68 | 29.12 | 6.26 | 20.64 | 4.80 |
| Kurnool | 41.25 | 8.49 | 35.55 | 5.14 | 18.19 | 3.51 |
| Kushi Nagar | 47.15 | 6.48 | 38.82 | 6.69 | 11.85 | 2.49 |
| Lakhimpur | 32.73 | 6.27 | 22.14 | 5.01 | 9.68 | 2.49 |
| Lakshadweep | 26.52 | 3.99 | 21.49 | 3.19 | 13.01 | 2.45 |
| Lalganj | 41.92 | 7.50 | 33.38 | 6.83 | 18.36 | 4.22 |
| Latur | 33.35 | 5.53 | 33.56 | 6.57 | 19.96 | 5.01 |
| Leh (Ladakh) | 29.75 | 6.27 | 17.11 | 4.11 | 11.23 | 3.37 |
| Lohardaga | 44.13 | 5.25 | 54.23 | 6.75 | 37.83 | 8.43 |
| Lucknow | 41.24 | 4.05 | 39.36 | 3.83 | 14.69 | 3.76 |
| Ludhiana | 24.27 | 5.37 | 18.60 | 3.71 | 14.01 | 3.93 |
| Machhlishahr | 48.62 | 6.97 | 32.36 | 4.16 | 16.76 | 4.14 |
| Machilipatnam | 26.00 | 2.97 | 29.48 | 4.35 | 17.49 | 3.79 |
| Madha | 26.33 | 3.94 | 28.62 | 4.04 | 22.20 | 4.24 |
| Madhepura | 50.05 | 7.79 | 45.30 | 7.14 | 20.81 | 5.79 |
| Madhubani | 46.88 | 9.87 | 38.41 | 6.82 | 17.23 | 4.22 |
| Madurai | 23.71 | 3.39 | 25.67 | 4.40 | 26.85 | 5.66 |
| Mahabubabad | 25.90 | 3.58 | 27.49 | 2.63 | 14.90 | 2.73 |
| Maharajganj | 41.93 | 6.32 | 39.45 | 7.40 | 16.63 | 4.30 |
| Maharajganj | 53.47 | 5.60 | 27.06 | 4.94 | 10.68 | 2.98 |
| Mahasamund | 39.88 | 5.84 | 39.53 | 5.26 | 22.23 | 4.83 |
| Mahbubnagar | 29.56 | 5.15 | 25.02 | 4.36 | 16.68 | 3.09 |
| Mahesana | 41.98 | 6.34 | 43.23 | 4.78 | 23.53 | 4.79 |
| Mainpuri | 48.23 | 7.59 | 38.12 | 5.80 | 12.81 | 2.66 |
| Malappuram | 24.84 | 4.98 | 13.09 | 1.64 | 13.76 | 2.31 |
| Maldah Dakshin | 34.23 | 7.85 | 37.80 | 6.03 | 23.69 | 9.61 |
| Maldah Uttar | 39.32 | 5.47 | 37.10 | 4.81 | 21.25 | 4.50 |
| Malkajgiri | 25.52 | 4.24 | 23.39 | 3.75 | 14.65 | 2.61 |
| Mandi | 21.50 | 3.48 | 30.05 | 5.59 | 32.13 | 8.07 |
| Mandsaur | 34.71 | 5.07 | 46.76 | 6.46 | 29.48 | 6.13 |
| Mandya | 23.87 | 3.46 | 49.01 | 6.63 | 25.06 | 5.22 |
| Mangaldoi | 36.22 | 6.84 | 29.60 | 6.27 | 16.54 | 4.26 |
| Mathura | 39.93 | 6.48 | 32.38 | 5.77 | 11.80 | 2.93 |
| Mathurapur | 27.98 | 4.82 | 30.09 | 4.30 | 17.92 | 3.30 |
| Maval | 32.58 | 5.45 | 35.67 | 5.72 | 27.85 | 4.74 |
| Mavelikkara | 16.26 | 2.41 | 18.59 | 2.41 | 15.11 | 3.39 |
| Mayiladuthurai | 24.46 | 3.58 | 23.67 | 3.41 | 18.89 | 3.13 |
| Mayurbhanj | 38.81 | 6.52 | 38.20 | 6.35 | 16.74 | 4.25 |
| Medak | 30.35 | 4.03 | 34.29 | 5.43 | 20.79 | 4.20 |
| Medinipur | 23.52 | 2.67 | 34.24 | 4.84 | 16.90 | 3.07 |
| Meerut | 35.09 | 6.93 | 44.62 | 6.27 | 17.06 | 3.61 |
| Mirzapur | 48.91 | 6.77 | 45.48 | 8.58 | 18.00 | 4.14 |
| Misrikh | 50.22 | 6.09 | 40.78 | 6.27 | 11.26 | 2.28 |
| Mizoram | 28.46 | 5.82 | 12.80 | 2.90 | 7.33 | 1.96 |
| Mohanlalganj | 39.84 | 5.24 | 39.07 | 6.14 | 28.58 | 7.10 |
| Moradabad | 39.82 | 7.82 | 36.55 | 6.62 | 17.18 | 4.27 |
| Morena | 48.38 | 8.85 | 53.16 | 6.65 | 28.27 | 6.25 |
| Mumbai North | 27.13 | 4.45 | 34.75 | 5.29 | 23.92 | 4.62 |
| Mumbai North-Central | 31.48 | 3.99 | 31.98 | 4.42 | 18.70 | 2.55 |
| Mumbai North-East | 24.23 | 3.15 | 23.64 | 3.05 | 22.65 | 5.48 |
| Mumbai North-West | 26.76 | 3.23 | 31.26 | 4.28 | 30.56 | 6.04 |
| Mumbai South | 26.49 | 1.75 | 26.67 | 2.68 | 24.54 | 3.30 |
| Mumbai South-Central | 34.08 | 5.09 | 34.76 | 5.84 | 22.32 | 3.66 |
| Munger | 45.63 | 7.50 | 43.97 | 6.21 | 20.16 | 6.85 |
| Murshidabad | 33.07 | 5.95 | 31.32 | 7.58 | 14.85 | 2.70 |
| Muzaffarnagar | 37.89 | 6.40 | 38.88 | 7.21 | 26.36 | 6.40 |
| Muzaffarpur | 45.85 | 6.75 | 42.43 | 5.98 | 17.02 | 2.95 |
| Mysore | 28.22 | 4.23 | 33.65 | 4.13 | 21.69 | 4.30 |
| Nabarangapur | 42.61 | 6.33 | 17.01 | 2.58 | 11.71 | 2.03 |
| Nagaland | 27.66 | 5.40 | 16.06 | 3.34 | 10.94 | 3.12 |
| Nagaon | 34.77 | 7.62 | 24.14 | 5.52 | 10.52 | 2.44 |
| Nagappattinam | 27.11 | 4.77 | 19.14 | 2.90 | 13.66 | 2.83 |
| Nagarkurnool | 35.83 | 6.81 | 35.31 | 6.90 | 18.21 | 4.28 |
| Nagaur | 38.41 | 6.35 | 21.61 | 3.77 | 13.13 | 3.00 |
| Nagina | 42.03 | 7.60 | 39.48 | 6.40 | 17.37 | 3.40 |
| Nagpur | 25.25 | 4.23 | 23.36 | 3.55 | 18.78 | 3.14 |
| Nainital - Udhamsingh Nagar | 33.10 | 6.12 | 24.94 | 4.46 | 20.84 | 5.45 |
| Nalanda | 52.99 | 7.49 | 46.50 | 7.81 | 21.33 | 5.40 |
| Nalgonda | 28.71 | 5.81 | 31.71 | 5.25 | 21.02 | 5.34 |
| Namakkal | 25.66 | 5.00 | 21.67 | 3.78 | 17.41 | 3.16 |
| Nanded | 40.89 | 7.34 | 35.77 | 6.63 | 19.72 | 3.65 |
| Nandurbar | 44.69 | 6.90 | 53.26 | 6.17 | 33.57 | 6.11 |
| Nandyal | 36.47 | 6.40 | 33.51 | 4.52 | 14.76 | 2.51 |
| Narasaraopet | 30.95 | 4.22 | 32.17 | 4.70 | 13.55 | 2.21 |
| Narsapuram | 28.32 | 4.25 | 32.69 | 4.21 | 16.52 | 1.83 |
| Nashik | 35.65 | 4.28 | 32.70 | 6.59 | 26.37 | 5.81 |
| Navsari | 34.85 | 5.44 | 47.64 | 6.32 | 29.15 | 6.04 |
| Nawada | 48.87 | 8.02 | 47.31 | 6.44 | 21.88 | 5.50 |
| Nellore | 24.56 | 3.83 | 27.30 | 5.78 | 16.82 | 3.93 |
| New Delhi | 27.69 | 4.24 | 27.65 | 3.41 | 25.57 | 5.31 |
| Nilgiris | 31.30 | 4.88 | 25.20 | 3.46 | 20.05 | 4.17 |
| Nizamabad | 29.67 | 5.11 | 34.52 | 4.17 | 18.75 | 2.90 |
| North East Delhi | 26.71 | 4.14 | 22.82 | 3.22 | 22.76 | 5.36 |
| North Goa | 23.33 | 3.56 | 23.49 | 3.64 | 32.87 | 5.34 |
| North West Delhi | 38.28 | 3.51 | 33.16 | 4.26 | 20.30 | 3.62 |
| Ongole | 29.99 | 6.56 | 33.42 | 4.94 | 16.37 | 3.40 |
| Osmanabad | 38.53 | 4.71 | 39.41 | 4.69 | 20.67 | 4.82 |
| Outer Manipur | 32.89 | 6.74 | 48.53 | 6.35 | 27.76 | 6.06 |
| Palakkad | 20.32 | 4.29 | 13.93 | 1.61 | 14.53 | 2.74 |
| Palamu | 44.78 | 6.71 | 46.87 | 6.27 | 29.00 | 6.46 |
| Palghar | 37.86 | 5.29 | 38.17 | 3.52 | 31.60 | 5.59 |
| Pali | 40.11 | 6.35 | 37.99 | 5.86 | 25.03 | 5.19 |
| Panch Mahals | 41.08 | 5.35 | 45.00 | 5.83 | 26.44 | 5.62 |
| Parbhani | 44.48 | 6.56 | 40.72 | 5.07 | 19.09 | 4.03 |
| Pashchim Champaran | 42.57 | 5.33 | 37.96 | 7.09 | 17.36 | 3.84 |
| Pataliputra | 47.24 | 8.50 | 45.38 | 6.90 | 26.03 | 6.71 |
| Patan | 37.82 | 5.16 | 40.06 | 5.80 | 30.93 | 5.86 |
| Pathanamthitta | 17.46 | 3.04 | 18.46 | 2.54 | 20.29 | 3.25 |
| Patiala | 21.03 | 3.77 | 27.64 | 6.15 | 19.22 | 5.02 |
| Patna Sahib | 39.67 | 7.92 | 41.43 | 6.99 | 25.85 | 7.11 |
| Peddapalle | 23.18 | 3.94 | 23.58 | 3.26 | 16.97 | 3.05 |
| Perambalur | 26.13 | 5.43 | 22.06 | 4.43 | 17.26 | 4.10 |
| Phulpur | 42.65 | 6.46 | 47.64 | 7.77 | 22.57 | 5.32 |
| Pilibhit | 49.26 | 8.21 | 29.84 | 4.79 | 13.14 | 2.71 |
| Pollachi | 23.41 | 3.59 | 28.28 | 4.01 | 18.66 | 3.16 |
| Ponnani | 17.01 | 2.98 | 12.60 | 2.64 | 5.86 | 1.25 |
| Porbandar | 25.03 | 3.71 | 50.31 | 6.74 | 36.88 | 7.07 |
| Pratapgarh | 40.10 | 7.17 | 51.04 | 7.71 | 25.26 | 7.28 |
| Puducherry | 25.16 | 4.35 | 24.23 | 4.71 | 17.09 | 3.95 |
| Pune | 27.16 | 6.08 | 32.20 | 2.67 | 27.67 | 4.29 |
| Purba Champaran | 49.41 | 7.32 | 39.73 | 5.38 | 17.12 | 3.36 |
| Puri | 22.86 | 4.48 | 41.90 | 5.82 | 24.91 | 4.84 |
| Purnia | 51.38 | 6.23 | 44.01 | 6.69 | 19.43 | 4.01 |
| Puruliya | 40.29 | 7.41 | 53.97 | 6.44 | 31.76 | 6.48 |
| Rae Bareli | 37.07 | 7.24 | 42.34 | 7.57 | 21.87 | 6.28 |
| Raichur | 44.11 | 6.66 | 42.20 | 7.65 | 23.20 | 5.11 |
| Raiganj | 37.07 | 7.77 | 32.50 | 6.19 | 14.83 | 3.39 |
| Raigarh | 35.99 | 5.93 | 35.54 | 5.65 | 17.93 | 4.34 |
| Raigarh | 27.36 | 3.80 | 31.45 | 4.80 | 21.59 | 4.66 |
| Raipur | 36.71 | 6.27 | 34.00 | 5.98 | 15.79 | 3.85 |
| Rajahmundry | 27.36 | 4.55 | 23.34 | 3.51 | 16.46 | 4.50 |
| Rajampet | 32.06 | 5.10 | 34.90 | 4.07 | 15.14 | 3.58 |
| Rajgarh | 40.76 | 7.60 | 35.23 | 4.79 | 18.99 | 4.72 |
| Rajkot | 32.61 | 4.80 | 44.25 | 4.84 | 23.83 | 5.01 |
| Rajmahal | 49.52 | 8.14 | 48.32 | 6.43 | 30.08 | 6.31 |
| Rajnandgaon | 44.25 | 8.58 | 37.36 | 7.54 | 17.10 | 4.75 |
| Rajsamand | 37.06 | 6.27 | 42.17 | 6.31 | 28.57 | 6.13 |
| Ramanathapuram | 23.30 | 4.38 | 22.57 | 3.83 | 15.15 | 3.30 |
| Rampur | 44.79 | 6.56 | 43.78 | 5.78 | 26.04 | 5.18 |
| Ramtek | 35.84 | 5.57 | 40.36 | 5.38 | 26.84 | 4.21 |
| Ranaghat | 26.39 | 2.21 | 19.94 | 2.13 | 13.55 | 2.51 |
| Ranchi | 37.06 | 6.23 | 49.85 | 6.49 | 33.11 | 7.59 |
| Ratlam | 46.81 | 8.15 | 36.57 | 5.33 | 17.68 | 3.48 |
| Ratnagiri - Sindhudurg | 30.51 | 4.05 | 31.37 | 3.14 | 21.68 | 3.10 |
| Raver | 29.49 | 4.95 | 34.51 | 4.72 | 26.53 | 4.03 |
| Rewa | 40.91 | 6.36 | 38.57 | 5.70 | 26.35 | 4.49 |
| Robertsganj | 45.21 | 6.73 | 45.64 | 7.05 | 18.47 | 4.95 |
| Rohtak | 29.05 | 5.64 | 24.66 | 4.53 | 16.30 | 4.15 |
| Sabar Kantha | 48.07 | 6.53 | 46.04 | 5.82 | 19.61 | 5.29 |
| Sagar | 40.11 | 5.92 | 36.17 | 5.51 | 18.97 | 4.03 |
| Saharanpur | 34.38 | 6.17 | 22.54 | 4.16 | 10.92 | 2.51 |
| Salem | 26.06 | 5.19 | 22.25 | 3.62 | 19.69 | 4.39 |
| Salempur | 39.65 | 7.08 | 33.96 | 4.33 | 15.43 | 2.68 |
| Samastipur | 51.70 | 7.58 | 42.34 | 8.18 | 17.78 | 4.21 |
| Sambalpur | 34.00 | 5.77 | 37.86 | 5.60 | 22.31 | 4.54 |
| Sambhal | 45.50 | 6.70 | 38.74 | 6.94 | 17.77 | 3.86 |
| Sangli | 28.18 | 5.82 | 28.99 | 4.66 | 17.83 | 2.76 |
| Sangrur | 25.96 | 4.88 | 22.10 | 3.68 | 13.66 | 2.64 |
| Sant Kabir Nagar | 49.46 | 6.86 | 37.18 | 6.21 | 14.67 | 3.23 |
| Sant Ravi Das Nagar (Bhadohi) | 50.00 | 6.55 | 37.90 | 6.03 | 18.20 | 3.78 |
| Saran (Chhapra) | 47.31 | 7.37 | 41.25 | 7.32 | 17.80 | 4.25 |
| Sasaram | 52.45 | 6.66 | 48.14 | 6.42 | 21.56 | 6.41 |
| Satara | 24.28 | 3.22 | 29.96 | 3.84 | 22.56 | 4.59 |
| Satna | 40.36 | 6.62 | 35.11 | 5.76 | 21.78 | 4.62 |
| Secunderabad | 17.92 | 2.42 | 19.38 | 2.96 | 14.94 | 2.62 |
| Shahdol | 36.76 | 6.01 | 43.43 | 7.29 | 24.21 | 5.79 |
| Shahjahanpur | 48.21 | 6.83 | 52.19 | 5.75 | 17.91 | 3.47 |
| Sheohar | 52.96 | 6.84 | 43.17 | 7.61 | 14.62 | 3.41 |
| Shillong | 48.74 | 7.99 | 29.97 | 5.77 | 12.45 | 4.26 |
| Shimla | 25.80 | 4.72 | 16.00 | 3.00 | 13.45 | 3.09 |
| Shimoga | 30.54 | 3.94 | 25.68 | 3.57 | 18.10 | 2.80 |
| Shirdi | 36.67 | 4.94 | 32.65 | 4.45 | 22.52 | 5.08 |
| Shirur | 31.90 | 5.48 | 27.16 | 3.38 | 16.48 | 2.87 |
| Shivaganga | 22.54 | 3.58 | 27.01 | 5.38 | 14.22 | 3.93 |
| Shrawasti | 60.92 | 6.64 | 38.82 | 6.28 | 13.57 | 3.01 |
| Shrirampur | 29.68 | 5.75 | 29.81 | 4.80 | 16.74 | 3.63 |
| Sidhi | 39.89 | 6.75 | 48.05 | 6.51 | 30.77 | 6.67 |
| Sikar | 29.41 | 5.81 | 36.56 | 6.85 | 21.03 | 4.75 |
| Sikkim | 28.70 | 4.62 | 26.61 | 3.94 | 11.72 | 2.09 |
| Silchar | 34.20 | 5.56 | 34.37 | 5.74 | 27.55 | 6.21 |
| Singhbhum | 53.40 | 6.71 | 48.00 | 6.50 | 26.05 | 6.13 |
| Sirsa | 30.22 | 5.50 | 35.16 | 6.72 | 21.48 | 5.22 |
| Sitamarhi | 58.56 | 6.28 | 48.88 | 5.79 | 15.30 | 2.80 |
| Sitapur | 53.84 | 8.20 | 41.39 | 6.65 | 13.10 | 3.56 |
| Siwan | 37.62 | 6.39 | 29.90 | 5.56 | 14.32 | 3.04 |
| Solapur | 30.96 | 5.18 | 32.91 | 4.63 | 23.38 | 3.63 |
| Sonipat | 34.60 | 7.24 | 25.68 | 4.89 | 22.07 | 7.25 |
| South Delhi | 30.34 | 4.51 | 28.42 | 4.12 | 20.19 | 3.31 |
| South Goa | 17.21 | 2.41 | 20.77 | 3.05 | 14.58 | 3.09 |
| Srikakulam | 29.75 | 4.23 | 31.02 | 5.40 | 15.94 | 3.09 |
| Srinagar | 24.34 | 5.02 | 10.00 | 1.85 | 7.23 | 1.78 |
| Sriperumbudur | 24.03 | 4.15 | 33.71 | 5.62 | 30.75 | 9.56 |
| Sultanpur | 42.39 | 5.92 | 37.53 | 6.60 | 17.44 | 5.01 |
| Sundargarh | 35.46 | 5.62 | 40.31 | 5.55 | 27.70 | 5.76 |
| Supaul | 46.11 | 7.44 | 44.47 | 7.17 | 23.36 | 5.98 |
| Surat | 25.90 | 4.09 | 31.52 | 3.97 | 22.17 | 3.98 |
| Surendranagar | 44.20 | 5.24 | 33.41 | 4.87 | 20.53 | 4.39 |
| Surguja | 31.56 | 4.97 | 34.04 | 5.46 | 21.16 | 4.64 |
| Tamluk | 26.16 | 4.60 | 30.49 | 5.29 | 23.79 | 5.54 |
| Tehri Garhwal | 32.01 | 5.41 | 25.74 | 4.81 | 15.69 | 4.46 |
| Tenkasi | 27.74 | 4.62 | 20.04 | 3.08 | 15.92 | 2.97 |
| Tezpur | 31.05 | 5.31 | 25.55 | 4.49 | 16.61 | 5.84 |
| Thane | 28.27 | 4.36 | 32.64 | 4.20 | 25.24 | 3.94 |
| Thanjavur | 23.49 | 3.75 | 19.51 | 3.22 | 15.75 | 3.35 |
| Theni | 26.66 | 4.32 | 22.18 | 3.22 | 18.90 | 3.76 |
| Thiruvananthapuram | 17.77 | 2.64 | 16.97 | 4.79 | 10.94 | 3.30 |
| Thoothukkudi | 21.44 | 3.38 | 22.44 | 4.81 | 23.51 | 6.29 |
| Thrissur | 18.40 | 2.41 | 15.91 | 2.91 | 11.49 | 1.68 |
| Tikamgarh | 46.48 | 7.24 | 44.15 | 6.12 | 20.59 | 4.21 |
| Tiruchirappalli | 28.14 | 5.56 | 22.56 | 4.11 | 18.96 | 3.84 |
| Tirunelveli | 27.81 | 3.34 | 22.40 | 3.20 | 14.24 | 2.48 |
| Tirupati | 29.84 | 4.66 | 27.04 | 5.14 | 13.65 | 2.94 |
| Tiruppur | 23.78 | 5.13 | 24.55 | 3.76 | 13.98 | 1.89 |
| Tiruvallur | 32.03 | 5.06 | 27.89 | 3.79 | 20.66 | 4.33 |
| Tiruvannamalai | 27.59 | 3.79 | 25.48 | 5.68 | 19.84 | 6.38 |
| Tonk - Sawai Madhopur | 34.69 | 6.12 | 31.28 | 6.11 | 18.52 | 5.16 |
| Tripura East | 26.58 | 4.84 | 19.33 | 3.14 | 19.67 | 3.68 |
| Tripura West | 20.20 | 3.20 | 25.35 | 4.65 | 16.43 | 3.42 |
| Tumkur | 28.55 | 3.30 | 28.10 | 3.48 | 17.66 | 2.85 |
| Tura | 26.39 | 5.35 | 23.38 | 4.92 | 20.74 | 5.64 |
| Udaipur | 44.46 | 8.65 | 49.86 | 7.59 | 31.26 | 6.81 |
| Udhampur | 32.33 | 6.15 | 52.42 | 5.76 | 31.65 | 6.45 |
| Udupi Chikmagalur | 23.68 | 2.97 | 25.39 | 3.51 | 23.42 | 4.73 |
| Ujiapur | 49.12 | 8.56 | 43.39 | 6.92 | 16.90 | 3.44 |
| Ujjain | 36.63 | 6.31 | 39.33 | 6.01 | 21.77 | 4.45 |
| Uluberiya | 31.10 | 4.30 | 30.06 | 4.18 | 17.83 | 4.28 |
| Unnao | 46.68 | 6.60 | 34.94 | 4.47 | 16.31 | 2.59 |
| Uttara Kannada | 38.01 | 5.06 | 26.76 | 3.75 | 16.12 | 2.62 |
| Vadakara | 18.97 | 2.70 | 19.34 | 2.98 | 12.66 | 2.14 |
| Vadodara | 35.70 | 4.41 | 37.78 | 5.61 | 21.27 | 4.00 |
| Vaishali | 48.01 | 7.19 | 40.43 | 7.61 | 16.74 | 4.49 |
| Valmiki Nagar | 43.93 | 7.10 | 41.85 | 6.78 | 21.66 | 5.38 |
| Valsad | 43.04 | 6.93 | 38.68 | 4.77 | 29.03 | 4.31 |
| Varanasi | 41.36 | 6.80 | 46.86 | 7.09 | 26.59 | 5.63 |
| Vellore | 27.76 | 5.42 | 17.66 | 2.59 | 17.88 | 3.64 |
| Vidisha | 40.02 | 7.10 | 48.30 | 7.37 | 27.61 | 6.93 |
| Vijayawada | 25.18 | 2.37 | 23.13 | 2.71 | 13.51 | 2.15 |
| Viluppuram | 29.82 | 5.32 | 30.47 | 4.71 | 17.84 | 2.68 |
| Virudunagar | 27.82 | 4.70 | 19.99 | 2.35 | 15.54 | 2.71 |
| Visakhapatnam | 25.32 | 3.34 | 27.03 | 3.77 | 17.07 | 3.66 |
| Vizianagaram | 35.03 | 5.58 | 32.99 | 4.52 | 13.67 | 3.23 |
| Warangal | 24.97 | 4.22 | 29.51 | 5.03 | 18.81 | 3.46 |
| Wardha | 33.62 | 5.01 | 38.32 | 5.42 | 23.92 | 4.23 |
| Wayanad | 24.47 | 4.01 | 15.68 | 2.33 | 25.56 | 6.16 |
| West Delhi | 25.52 | 3.99 | 17.56 | 2.16 | 15.02 | 2.84 |
| Yavatmal - Washim | 41.72 | 7.30 | 43.29 | 5.96 | 26.55 | 5.34 |
| Zahirabad | 35.19 | 5.37 | 35.44 | 5.43 | 20.69 | 4.13 |
